# Analysis of Demographic and Practice Characteristics of Psychiatrists in Three Canadian Provinces: Analyse des caractéristiques démographiques et de la pratique des psychiatres dans trois provinces canadiennes

**DOI:** 10.1177/07067437251359183

**Published:** 2025-07-21

**Authors:** François Gallant, Ridhwana Kaoser, Sandra Peterson, Matt Dahl, Alison L. Park, James M. Bolton, Myriam Juda, Alan Katz, Jason Morrison, Benoit H. Mulsant, Philip G. Tibbo, Juveria Zaheer, Paul Kurdyak, M. Ruth Lavergne, David Rudoler

**Affiliations:** 1 Department of Family Medicine, 3688 Dalhousie University, Halifax, Canada; 2 Vitalité Health Network, Moncton, New Brunswick, Canada; 3 Faculty of Health Sciences, Simon Fraser University at Harbour Centre, Vancouver, British Columbia, Canada; 4 Centre for Health Services and Policy Research, School of Population and Public Health, 8166 The University of British Columbia, Vancouver, British Columbia, Canada; 5 Manitoba Centre for Health Policy, 8664 University of Manitoba, Manitoba, Canada; 6 ICES, Toronto, Ontario, Canada; 7 Department of Psychiatry, 8664 University of Manitoba, Manitoba, Canada; 8 Department of Psychology, 1763 Simon Fraser University, Burnaby, British Columbia, Canada; 9 Department of Community Health Sciences, Rady Faculty of Health Sciences, 8664 University of Manitoba, Winnipeg, Manitoba, Canada; 10 Department of Family Medicine, Rady Faculty of Health Sciences, 8664 University of Manitoba, Winnipeg, Manitoba, Canada; 11 Department of Psychiatry, 3688 Dalhousie University, Halifax, Canada; 12 Centre for Addiction and Mental Health, Toronto, Ontario, Canada; 13 Department of Psychiatry, Temerty Faculty of Medicine, University of Toronto, Ontario, Canada; 14 Faculty of Health Sciences, 85458 Ontario Tech University, Oshawa, Ontario, Canada; 15 Ontario Shores Centre for Mental Health Sciences, Whitby, Ontario, Canada

**Keywords:** mental health services, psychiatric workforce, clinical complexity, physician practice patterns, physician demographics

## Abstract

**Objective:**

To describe demographic and practice characteristics of psychiatrists in British Columbia (BC), Manitoba (MB) and Ontario (ON) and explore how practice characteristics change by psychiatrist sex/gender and years since medical school graduation.

**Method:**

We conducted a repeated cross-sectional study of all practising psychiatrists who had patient interactions and submitted billings from the fiscal years (FY) 2012/2013 to 2021/2022 using linked administrative data in BC, MB and ON. Psychiatrist demographic variables included age, sex/gender, years since medical school graduation and their practice location. Psychiatrist practice characteristics included visit and patient volume, service settings and patient diagnoses. We used measures of central tendency to describe demographic and practice characteristics and quantify change over time using percentage change.

**Results:**

The number of psychiatrists increased from 2012/2013 to 2021/2022 (percentage change, BC: 15.4%, MB: 20.0%, ON: 11.8%) and kept up with population increases, shown by stable per-capita supply of psychiatrists. The median age of psychiatrists in all provinces decreased over the study period. The percentage of female psychiatrists in practice increased in all provinces, but more in BC and ON than in MB. On average, psychiatrists are seeing a greater number of patients per year in 2021/2022 than in 2012/2013 (percentage change, BC: 14.6%, MB: 6.5%, ON: 11.1%), and more than half of patients are seen, on average, for one or two visits in all three provinces. More patients receive substance use and psychosis diagnoses over the 10-year study.

**Conclusions:**

During the past decade, psychiatric practice characteristics show modest changes despite changing psychiatrist demographics and subtle shift towards more consultative practices. While provinces demonstrated similar trends, differences underscore the importance of conducting pan-Canadian analyses to highlight particularities in workforce patterns.

**Plain Language Summary Title:**

Describing 10-year change in psychiatrists’ demographics and practice characteristics in British Columbia, Manitoba and Ontario

## Plain Language Summary

We described psychiatrist demographic characteristics (age, sex/gender, years since medical school graduation), and practice characteristics (number of visits per year, number of patients per year, different patient diagnoses) in British Columbia, Manitoba and Ontario to see how patterns changed over the past 10 years.

We found that characteristics of psychiatrists have changed: psychiatrists are on average younger, and there are more female psychiatrists in practice. Psychiatrists are seeing more patients per year than they did in 2012, with most patients having only one or two visits. More patients are being diagnosed with substance use disorders and psychosis diagnoses compared to 10 years ago. We also note a shift towards virtual care.

Even though the characteristics of psychiatrists in practice have changed, the way they practice has mostly remained similar. This study also shows that while provinces show similar trends, some differences between provinces suggest that more studies comparing multiple provinces are needed.

## Résumé

**Objectif:**

Décrire les caractéristiques démographiques et de pratique des psychiatres en Colombie-Britannique (C.-B.), au Manitoba (Man.) et en Ontario (Ont.) et explorer la façon dont les caractéristiques de la pratique changent selon le sexe/genre du psychiatre et les années depuis l’obtention de son diplôme en médecine.

**Méthode:**

Nous avons mené une étude transversale répétée de tous les psychiatres actifs qui ont eu des interactions avec des patients et soumis des factures pour les exercices financiers de 2012–2013 à 2021–2022 à l’aide de données administratives en Colombie-Britannique, au Manitoba et en Ontario. Les variables démographiques des psychiatres comprenaient l’âge, le sexe/le genre, le nombre d’années depuis l’obtention du diplôme d’études en médecine et le lieu de pratique. Les caractéristiques de la pratique des psychiatres comprenaient les visites et le volume de patients, les contextes de service et le diagnostic des patients. Nous avons utilisé des mesures de tendance centrale pour décrire les caractéristiques démographiques et de la pratique et quantifier le changement au fil du temps en utilisant le pourcentage de changement.

**Résultats:**

Le nombre de psychiatres a augmenté de 2012–2013 à 2021–2022 (variation en pourcentage, C.-B. : 15,4%, Man. : 20,0%, Ont., 11,8%) et a suivi l’accroissement démographique, comme en témoigne le nombre stable de psychiatres par habitant. L’âge médian des psychiatres dans toutes les provinces a baissé au cours de la période à l’étude. Le pourcentage de femmes psychiatres en exercice a augmenté dans toutes les provinces, mais plus en Colombie-Britannique et en Ontario qu’au Manitoba. En moyenne, les psychiatres voient un plus grand nombre de patients par année en 2021–2022 qu’en 2012–2013 (variation en pourcentage, C.-B. : 14,6%, Man. : 6,5%, Ont. : 11,1%), et plus de la moitié des patients sont vus, en moyenne, pour une ou deux visites dans les trois provinces. Un plus grand nombre de patients a reçu un diagnostic de toxicomanie et de psychose au cours de l’étude de 10 ans.

**Conclusions:**

Au cours de la dernière décennie, les caractéristiques de la pratique psychiatrique ont montré des changements modestes malgré les variations dans les caractéristiques démographiques des psychiatres et un virage subtil vers des pratiques plus consultatives. Bien que les provinces aient montré des tendances semblables, les différences soulignent l’importance de mener des analyses pancanadiennes pour mettre en évidence les particularités des tendances relatives à la main-d’œuvre.

## Introduction

As approximately five million Canadians now report significant symptoms of mental illness, understanding how the psychiatric workforce is evolving to respond to changing needs is of urgent importance.^1–4^ Although family physicians and community services that include a range of service providers often represent the first point of contact for mental health treatment or referral to other mental health services,^5^ these points of contact often lack resources or supports to adequately address serious mental illness.^6^

Psychiatry plays a critical role within mental health services,^6,7^ but access has been a longstanding issue.^2^ Estimates suggest 13.1 psychiatrists per 100,000 population are practising in Canada,^8^ with evident variation in psychiatrist supply across geographic areas.^8,9^ Over and above per-capita supply, understanding practice characteristics such as practice location (e.g., most psychiatrists work in large urban centres),^10,11^ practice roster (e.g., some see very few new patients per year),^10,11^ and sub-specialisation (e.g., addiction psychiatry, youth psychiatry, etc.), may help address access gaps.^2^ Therefore, understanding their practice characteristics and changes in these characteristics over time is crucial for workforce planning and for developing policies and processes that ensure timely and appropriate patient access to mental health care. Data characterising the psychiatric workforce from Ontario have been published,^10–12^ but comparable data from other Canadian provinces is limited. Data from Ontario (2003–2013) indicated that many psychiatrists were nearing retirement, male and practised predominantly in urban areas. Longitudinal analysis of these psychiatrists also highlighted stability in practice patterns.^10^

The degree to which these patterns have changed since 2013 is unknown and it is unclear if psychiatrists in other Canadian jurisdictions share similar practice patterns to psychiatrists in Ontario. Therefore, this analysis aims to provide a 10-year longitudinal examination of the psychiatric workforce in three provinces to harmonise and study pan-Canadian indicators of mental health care.^13^ Specifically, our objectives are to: describe (a) demographic and (b) practice characteristics of clinically active psychiatrists in British Columbia (BC), Manitoba (MB) and Ontario (ON) between 2012/2013 and 2021/2022 and (c) explore how practice characteristics changed by psychiatrist sex/gender and years since medical school graduation.

## Methods

We conducted a repeated cross-sectional study of practising psychiatrists and patient interactions from the fiscal years (FY) 2012/2013 to 2021/2022 using linked administrative data in BC, MB and ON. The study was approved by the Ontario Tech University review board (REB number: 16637), Simon Fraser University and University of British Columbia harmonised review boards (REB number: H21-03694), and the University of Manitoba Health review board [HREB number: HS25310 (H2022:010)].

### Data Sources

We used health administrative databases linked by Population Data BC, the Manitoba Centre for Health Policy and the Institute for Clinical Evaluative Sciences (ICES) (ON) to create consistent variable definitions (Table A1 in the supplemental material describes the datasets used; Table A2 in the supplemental material describes the variables used). Data from ON were linked using unique encoded identifiers and analysed at ICES.

### Participants and Study Variables

We included physicians who made at least one billing claim with a certified specialty of psychiatry. Their demographic characteristics include age, sex/gender, years since medical school graduation and their practice location. Practice location of psychiatrists was based on where the majority of their patient contacts resided, using Statistical Area Classification (SAC) type (Metropolitan, Other urban, or Rural). We also present number of psychiatrists per capita per year, calculated by dividing the total number of psychiatrists included in our analysis by the total population of each province per year. These values are stratified by SAC type for each year. Psychiatrists’ practice characteristics were obtained through billing data in each province: we describe the number of visits (total, inpatient and outpatient), the number of patients seen [total, inpatient, outpatient and new outpatients (e.g., patients not seen by the same psychiatrist in the previous two years in any setting in MB and ON, and previous 12 months in BC, when patients were not in the hospital)], and their number of billing days on an annual basis. We describe the volume of psychotherapy visits, virtual care visits and visits based on diagnosis codes billed (mental health, substance use, non-mental health and non-substance use). We also describe patient populations by describing visits, based on a validated algorithm,^14^ with those having a diagnosis of substance use disorder and those having a diagnosis of schizophrenia spectrum disorder (schizophrenia, schizoaffective, or other psychotic disorders) that were seen by any provider in outpatient, emergency department, or inpatient settings. We detail the number of virtual visits, as well as report on the proportion of outpatients seen for 1‒2, 3‒5, 6‒11, and 12 or more visits.

### Statistical Analysis

We describe psychiatrists’ demographic and practice characteristics using mean (SD), median [interquartile range (IQR)] and *n* (%) for all variables for each province. To quantify change over time, we computed percentage change between variables from FY2012/2013 to FY2021/2022. To provide insight into how practice changes across sex/gender, and years since medical school graduation, we plotted trends over time of the median number of total visits by sex/gender and years since graduation in each province. We also plotted median total visits per FY groupings, plotted against years since medical school graduation, grouped in 10-year bands. Given that we observed the full population of billed psychiatric services, inferential statistics are not reported. Data by FY and by province are presented in Tables A3‒A11 in supplemental materials.

## Results

### Demographic Characteristics

The overall number of psychiatrists from 2012/2013 to 2021/2022 has increased but the per-capita supply of psychiatrists has remained stable in BC and ON (16.0 psychiatrists per 100k population to 15.9; 14.7 to 14.8, respectively), and increased in MB (11.8 to 12.9; Table 1). Compared to 2012/2013, the median age of psychiatrists in all provinces was lower in 2021/2022 [median (IQR); 51 (43–60) BC, 50 (41–62) MB, 52 (42–65) ON]. The percentage of female psychiatrists in practice increased in all provinces, more in BC and ON than in MB. While more than 80% of psychiatrists work in metropolitan locations (over 90% in ON), we note increases in the per capita supply of psychiatrists working in rural locations across all three provinces between 2012/2013 and 2021/2022.

**Table 1.** Change in Demographic Characteristics of Psychiatrists Practising in British Columbia, Manitoba and Ontario in 2012/2013 and 2021/2022.

	British Columbia			Manitoba			Ontario		
FY	2012/2013	2021/2022	Change %	2012/2013	2021/2022	Change %	2012/2013	2021/2022	Change %
*n*	754	870	15.4	155	186	20.0	2092	2339	11.8
per capita (100 k)	16.0	15.9	−0.6	11.8	12.9	9.3	14.7	14.8	0.7
Age (years), mean (SD)	52.5 (11.7)	51.8 (11.8)	−1.4	53.0 (11.4)	52.2 (13.1)	−1.5	55.3 (13.4)	53.6 (14.0)	−3.2
Median (IQR)	52 (43–61)	51 (43–60)	−1.9	53.5 (44–61)	50 (41–62)	-6.5	56 (45–64)	52 (42–65)	-7.1
Sex/gender, *n* (%) female	295 (39.1)	392 (45.1)	32.9	50 (32.9)	59 (31.7)	18.0	814 (38.9)	1095 (46.8)	34.5
Years since MD grad mean (SD)	25.7 (12.3)	24.6 (12.3)	−4.1	26.0 (12.0)	27.5 (12.5)	6.0	28.7 (13.3)	27.8 (14.3)	-3.3
Median (IQR)	25 (16–34)	24 (14–34)	−4.0	27 (16.5–33)	26 (17–37)	-3.7	29 (18–38)	26 (15–39)	-10.3
Full-time, *n* (%)	527 (69.9)	609 (70.0)	15.6	108 (69.7)	130 (69.9)	20.4	1464 (70.0)	1637 (70.0)	11.8
Metropolitan area,^a^ *n* (%)	610 (80.9)	690 (79.5)	13.1	136 (87.7)	160 (86.0)	19.4	1950 (93.5)	2173 (93.1)	11.4
per capita (100 k)	19.3	18.6	−3.6	16.8	17.9	6.5	16.1	16.1	0
Other urban area,^b^ *n* (%)	124 (16.5)	151 (17.4)	21.8	13 (8.4)	13 (7.0)	0	90 (4.3)	103 (4.4)	11.4
per capita (100k)	13.6	18.6	36.8	9.3	8.5	-8.6	8.2	8.7	6.1
Rural area,^c^ *n* (%)	20 (2.7)	26–30 (3.0–3.4)	35.0	6 (3.9)	13 (7.0)	23.8	45 (2.2)	56–60 (2.4–2.6)	31.1
per capita (100k)	3.6	4.4	22	1.8	3.5	94.4	4.8	6.0	25
Unknown area, *n* (%)	0	≤5		0	0		7	≤5	

*Note*. FY = fiscal year; SD = standard deviation; IQR = interquartile range; MD = medical degree.

^a^ Metropolitan area > 100,000 total population.

^b^ Other urban area 10,000–100,000 total population.

^c^ Rural area < 10,000 total population;

### Practice Characteristics

Both mean and median visits per psychiatrist (including inpatient and outpatient visits) increased in BC and MB but decreased in ON (Table 2). Annual numbers per psychiatrist of unique individuals seen, new outpatients seen, and billing days have increased across all provinces. The proportion of patients seen for one or two outpatient visits increased in all provinces, with the largest increase in ON. The proportion of patients seen for 3‒5 and those seen 6‒11 has decreased in all provinces. The proportion of psychiatrists seeing fewer than 100 unique patients per year was unchanged or decreased slightly over time in all provinces.

**Table 2.** Practice Characteristics of Psychiatrists in British Columbia, Manitoba and Ontario in 2012/2013 and 2020/2021.

		British Columbia			Manitoba			ONTARIO		
FY		2012/2013	2021/2022	Change %	2012/2013	2021/2022	Change %	2012/2013	2021/2022	Change %
Total number of visits	Mean (SD)	1333.9 (1361.1)	1436.8 (1505.7)	7.7	1300.2 (1072.4)	1602.9 (1585.8)	23.3	1362.1 (1275.6)	1344.2 (1299.0)	−1.3
	Median (IQR)	1076.5 (517–1740)	1137 (588–1911)	5.6	1047 (449–1941)	1109 (481–2499)	5.9	1089.5 (502.5–1791.5)	1049 (482–1763)	–3.7
Total number of inpatient visits	Mean (SD)	389.9 (625.0)	409.7 (640.8)	5.1	337.0 (560.6)	486.9 (917.1)	44.5	311.6 (633.8)	295.9 (549.2)	–5.0
	Median (IQR)	45 (2–640)	53.5 (4–690)	18.9	28 (1–581)	32.5 (2–789)	16.1	10 (0–237)	17 (1–299)	70.0
Total number of outpatient visits	Mean (SD)	944.0 (1155.7)	1027.2 (1369.1)	8.8	963.2 (877.2)	1116.0 (1007.6)	15.9	1050.6 (1057.7)	1048.2 (1121.2)	–0.2
	Median (IQR)	737.5 (308–1280)	718.5 (332–1290)	–2.6	756 (280–1410)	834 (377–1529)	10.3	829.5 (341.5–1425)	805 (344–1406)	–3.0
Total number individuals seen (inpatient or outpatient)	Mean (SD)	273.5 (304.7)	313.4 (327.2)	14.6	241.5 (189.9)	257.2 (205.2)	6.5	292.2 (360.4)	324.4 (378.1)	11.0
	Median (IQR)	202.5 (103–365)	225.5 (103–422)	11.4	222 (70–373)	218.5 (93–386)	–1.6	185 (77–374)	215 (86–435)	16.2
Number of new outpatients (not seen in prior 12 m)	Mean (SD)	117.0 (212.0)	133.3 (197.4)	14.0	85.1 (78.3)	106.6 (111.3)	25.3	115.7 (146.0)	116.6 (153.3)	0.8
	Median (IQR)	76 (35–151)	80 (26–172)	5.3	63 (21–129)	81 (28–153)	28.6	65 (26–146.5)	69 (26–146)	6.2
% Patients seen for 1–2 outpatient visits	Mean (SD)	48.6 (23.8)	50.0 (26.8)	2.9	53.2 (26.6)	53.9 (27.3)	1.3	48.2 (25.5)	52.3 (27.1)	8.5
	Median (IQR)	47.2 (30.8–63.3)	49.3 (28.6–69.1)	4.5	54.2 (32.3–72.9)	54.6 (30.9–75.8)	0.7	47.2 (27.8–66.3)	54.6 (31.1–72.5)	15.7
% Patients seen for 3–5 outpatient visits	Mean (SD)	22.4 (11.0)	21.6 (13.2)	–3.6	16.3 (10.0)	14.3 (9.3)	–12.3	20.6 (11.8)	19.0 (12.0)	–7.8
	Median (IQR)	23.1 (14.8–29.2)	22.2 (12.0–29.6)	–3.9	15.6 (10.5–23.1)	13.5 (8.3–19.6)	–13.5	20.4 (12.4–27.6)	18.4 (10.2–26.5)	–9.8
% Patients seen for 6–11 outpatient visits	Mean (SD)	16.9 (11.4)	16.0 (13.2)	–5.3	14.6 (9.8)	14.3 (10.1)	–2.1	15.9 (11.6)	14.7 (12.6)	–7.5
	Median (IQR)	15.8 (8.1–24.6)	13.1 (5.3–24.7)	–17.1	14.1 (8.1–20.8)	14.0 (6.7–20.6)	–0.7	14.9 (7.3–22.8)	12.4 (5.0–21.1)	–16.8
% Patients seen for 12+ outpatient visits	Mean (SD)	12.1 (17.5)	12.5 (20.2)	3.3	15.9 (18.9)	17.5 (20.3)	10.1	15.3 (20.8)	14.0 (21.6)	–8.5
	Median (IQR)	4.7 (1.1–15.1)	3.5 (0.3–15.2)	–25.5	8.4 (2.9–24.7)	9.5 (3.5–24.1)	13.1	5.4 (0.7–22.2)	4.0 (0.6–17.5)	–25.9
Number of billing days	Mean (SD)	168.2 (68.5)	174.3 (74.8)	3.6	185.4 (84.2)	213.4 (100.3)	15.1	165.0 (71.5)	170.2 (76.5)	3.1
	Median (IQR)	181.5 (125–218)	190 (130–227)	4.7	212 (118–246)	228 (145–301)	7.6	181 (117–218)	186 (117–227)	2.8
Saw <100 patients/year	(*n*, %)	180 (23.9)	209 (24.0)	16.1	49 (31.6)	49 (26.3)	0	647 (30.9)	656 (28.1)	1.4

*Note*. FY = fiscal year; SD = standard deviation; IQR = interquartile range.

### Visit Volume by sex/Gender and Years Since Medical School Graduation

Changes in service volume over the course of psychiatrists’ careers are modest (supplemental data, Figure A1). Psychiatrists practising in BC and ON generally show an inverted-U shape pattern for median visit number, with psychiatrists between 20 and 30 years after medical school graduation having the highest visit number. For example, and on average, female psychiatrists in BC <10 years since MD school graduation offer a median of 740 annual visits, provide around 970 between 20 and 30 years in practice, and around 500 annual visits over 40 years in practice. However, there is very little variation when visits are averaged over two FYs, and psychiatrists are grouped by 10-year bands since medical school graduation (e.g., differences between groupings represent around 50 annual visits). Further, the plots of median visit volume by psychiatrist sex/gender and years since medical school graduation (≤15 years, 16–30years, and >30 years), show no marked change in practice volume (supplemental data, Figure A2). However, overall, female psychiatrists have a lower volume of visits than males in all three provinces.

### Psychiatric Services and Patient Population

The number of billings for virtual care increased dramatically since 2019/2020 (Table 3). Psychotherapy visits decreased in all provinces from 2012/2013 to 2021/2022, with the largest decrease in ON. Diagnostic codes assigned in billing and shadow-billing show an increase in the number of visits with diagnoses other than mental health or substance use, though the proportion of these visits remains modest (range 2% to 13% of total visits). The number of visits for patients treated for substance use disorders and schizophrenia, schizoaffective and psychotic disorders increased in all three provinces.

**Table 3.** Service Volume for Specific Diagnoses and Psychiatrist Patient Population in British Columbia, Manitoba and Ontario in 2012/2013 and 2020/2021.

		British Columbia			Manitoba			Ontario		
FY		2012/2013	2021/2022	Change %	2012/2013	2021/2022	Change %	2012/2013	2021/2022	Change %
Total number of psychotherapy visits^a^	Mean (SD)	927.3 (734.9)	839.9 (697.5)	−9.4	426.7 (470.7)	311.3 (340.2)	−27.0	706.1 (692.6)	374.7 (564.6)	−46.9
	Median (IQR)	775.0 (377.0–1320.0)	708.5 (304.0–1187.0)	–8.58	259.0 (84.0–564.0)	206.0 (80.0–420.0)	−20.5	516.0 (164.5–1057.5)	109.0 (4.0–532.0)	−78.9
Number of virtual visits	Mean (SD)	3.2 (30.5)	459.5 (588.4)	NA	0.3 (2.2)	514.3 (566.3)	NA	2.7 (19.6)	744.3 (912.3)	NA
	Median (IQR)	0 (0–0)	274.5 (27–675)	NA	0 (0–0)	341.5 (147–652)	NA	0 (0–0)	510 (131–1036)	NA
*Service volume for specific diagnoses*										
Number of mental health Dx visits	Mean (SD)	1087.5 (917.3)	1117.8 (1002.7)	2.8	1148.3 (1017.9)	1400.0 (1305.7)	21.9	1231.9 (1151.4)	1183.7 (1138.5)	−3.9
	Median (IQR)	890.5 (416–1514)	888 (407–1588)	–0.3	875 (355–1805)	957 (365–2288)	9.4	966 (420–1668.5)	926 (375–1629)	−4.1
Number of substance use Dx visits	Mean (SD)	84.2 (862.1)	117.2 (828.6)	39.1	14.4 (32.8)	9.9 (36.0)	−31.0	56.0 (506.5)	72.2 (561.4)	28.9
	Median (IQR)	0 (0–11)	0 (0–13)		3 (0–17)	0 (0–6)	−100.00	0 (0–11.5)	1 (0–20)	
Number of Non-MH Non-SU Dx visits	Mean (SD)	162.6 (432.6)	203.2 (566.0)	25.0	137.8 (203.4)	194.5 (503.1)	41.1	86.3 (233.8)	177.9 (351.61)	106.0
	Median (IQR)	27.5 (3–114)	25 (3–145)	–9.1	59 (10–163)	60 (12–181)	1.7	8 (0–69)	33 (1–195)	312.5
*Patient population*										
Number of visits for patients treated for SUD	Mean (SD)	214.0 (940.8)	306.9 (904.5)	43.4	65.3 (94.92)	138.4 (203.4)	111.9	166.8 (547.5)	226.1 (643.1)	35.5
	Median (IQR)	71.5 (17–210)	93.5 (20–341)	30.8	31 (6–89)	47.5 (8–167)	53.23	48 (13–155)	64 (15–224)	33.3
Number of visits for patients treated for schizophrenia, etc.	Mean (SD)	411.4 (542.4)	508.1 (716.7)	23.5	354.8 (546.3)	545.3 (793.7)	53.7	332.9 (555.7)	388.1 (596.8)	16.6
	Median (IQR)	182 (34–587)	225.5 (37–760)	23.9	85 (10–471)	151.5 (21–727)	78.2	95 (19–390.5)	120 (24–477)	26.3

*Note*. FY = fiscal year; Dx = diagnosis; SU = substance use; SUD= substance use disorder; SD = standard deviation; IQR = interquartile range; NA = percentage change not calculated.

^a^ Psychotherapy in ON may be underrepresented in ON FY2021/2022 due to billing code changes during the COVID-19 pandemic.

## Discussion

This analysis of practice characteristics of psychiatrists in three Canadian provinces over a decade shows similar levels of visit volumes despite changes in psychiatrist demographics. Psychiatrists are seeing a greater number of individual patients (e.g., percentage change over a decade 14.6% (BC), 6.5% (MB), 11.1% (ON)), with a higher proportion of patients seen for one or two visits, suggesting a shift towards increasing consultative practices. At the same time, psychiatrists are seeing more patients with substance use (percentage change over a decade: 43.4% (BC), 111.9% (MB), 35.5% (ON) and psychosis diagnoses: 23.5% (BC), 53.7% (MB), 16.6% (ON)). This analysis also highlights the importance of conducting pan-Canadian analyses and identifies important foci for future analyses given some notable variation in practice characteristics across provinces.

The number of practising psychiatrists has increased, but per-capita supply of psychiatrists has remained mostly stable. We note a decrease in the average age and an increase in the percentage of female psychiatrists, consistent with observations for other medical specialties.^15,16^ Female psychiatrists, despite earlier concerns,^10^ have not had a large impact on practice volumes. The slightly decreased volumes seen here might be offset by other evidence suggesting that female physicians spend more time and/or provider higher quality care to patients.^15,16^ In addition, visit volume by years since medical school graduation and physician sex/gender seems to change only modestly over psychiatrists’ careers. This is distinct from typical career trajectories seen in other medical specialities, showing more pronounced U-shaped patterns for visit volume, peaking around mid-career.^17,18^ This observation may require further study to better understand what shapes the career trajectories of psychiatrists and aid in future mental health workforce planning.

This analysis also characterised psychiatry patient populations by investigating billing codes reported by psychiatrists and diagnostic codes given to their patients from any health-care setting. Overall, we note an increase in the number of patients treated for substance use disorders and those treated for schizophrenia, schizoaffective and other psychotic disorders. This is not surprising given recent increases in the prevalence of substance use disorders^19^ and increased psychological distress in the wake of the COVID-19 pandemic,^20^ which may have been exacerbated among those with pre-existing mental health disorders.^19,21^ However, we also note an increase, on average, for mental health and substance use billing codes, as well as visits for non-mental health and non-substance use billing codes. Many of these patients could benefit from primary health care contacts within comprehensive collaborative care practices.^6,7^ As such, more resources should be made available to integrate psychiatrists and primary health teams.

Our data also suggest an increase in consultative practice models, rather than more sustained long-term follow-ups. For example, in BC and MB, we note an increase in the number of new outpatients accompanied by an increased proportion of patients seen once or twice within the year. This is consistent with consultative practices as defined by seeing a patient fewer than four visits.^11^ In our analysis, about half of the patients are seen once or twice per year, compared to data published in Ontario in 2009 when about half of patients were seen between four and 16 times.^11^ A lower number of consultations per psychiatrist might not adequately address mental health needs of clinically complex patients. However, if consultations occur in team-based settings (where the input of a psychiatrist is consolidated into treatment by a team), treatment efficacy might improve. While we are unable to test this hypothesis with our data, this analysis also highlights that stable or slowly increasing visit volume as a whole may not be sufficient to meet changing population needs. Therefore, models of health care delivery, where roles of providers, including psychiatrists, are optimised, warrant further thought.

Our data also document a substantial increase in the use of virtual care across all three provinces since 2019/2020. Virtual care is efficient to schedule, and psychiatrists can use this approach for clinical work that might not take a lot of time (e.g., refills/check-ins). In addition, virtual care is practical for patients (by alleviating the need to travel, find parking, or pay for public transit) and it may improve service utilisation for individuals with anxiety disorders or physical limitations who are not comfortable travelling the distance or waiting in a waiting room. Despite these advantages, virtual care may not be suitable for all patient populations, including unhoused individuals and those lacking access to technology (e.g., smartphone, reliable internet). Virtual care also provides limited access to critical interview information, such as critical physical cues such as changes in a patients’ body language or weight change indicative of metabolic syndrome due to medication. It has been shown to have similar outcomes to in-person consultations,^22^ making it an important and viable option for patient consultations in the future. Virtual care may improve access to psychiatric care as unplanned urgent visits can be delivered in a timely manner. However, differences in billing code requirements across provinces make direct inter-provincial comparisons on virtual care delivery difficult. For example, BC and MB have specific virtual psychotherapy codes whereas psychotherapy and virtual care are combined in ON.

Our analysis highlights substantial variability across provinces in aggregate billing data. For example, the median number of total inpatient visits per psychiatrist in Manitoba (FY2021/2022) was 32.5 (IQR: 2–789), compared to 17 (IQR: 1–299) in Ontario for the same FY. Past work suggests that practice styles of psychiatrists may help explain some of this variability. Specifically, using mixture modelling, Rudoler and colleagues^9^ identified that psychiatrists in Ontario can be categorised into one of three different practice styles.^12^ In addition, we note that supply of psychiatrists is lowest in MB and total service delivery per psychiatrist is higher in MB than in BC or ON. Future work should characterise practice styles in other Canadian provinces, as this work suggests that some variation in practice styles between provinces may be explained by some differences between provinces (e.g., in supply of psychiatrists or in compensation models). Identifying practice styles of psychiatrists is important because not all psychiatrists practice the same (e.g., general outpatient vs. sub-speciality), and practice styles are likely to vary geographically. Further, correctly identifying, and characterising sub-specialities will allow more in-depth understanding of practice characteristics, as patient populations across sub-specialities have different needs (e.g., pediatric psychiatry, geriatric psychiatry, etc.). Thus, identifying and understanding psychiatrists with different practice styles will make for more sophisticated health human resource planning.

Given our use of administrative (billing) data, some limitations must be considered when interpreting the data. Although we tried to maximise the identification of fee codes available to us to ensure representativeness of clinical activity, some fee codes may have been missed. Also, despite observing all psychiatric services billed under health insurance over a 10-year period, we are limited to patient contacts claimed or shadow-billed by psychiatrists and are not able to consider services provided by salaried psychiatrists if they do not shadow-bill. Past work from our research team shows that approximately 10% of psychiatrists were unaccounted for in all three provincial datasets.^9^ Since salaried psychiatrists in all three provinces would have similar incentives to report clinical activity in the billing data, we suspect that the data are similarly biased across provinces. Also, given our use of billing data, we are reporting on the use of psychiatric services rather than on population needs and unmet needs. Further, the specificity of billing codes in each province limits our interpretation of certain findings. For example, since virtual care and psychotherapy in ON are included in the same billing code, we are unable to comment on whether psychotherapy provision has actually decreased in ON or if this is an artefact of billing codes introduced during the COVID-19 pandemic. In addition, billing conventions of provinces may preclude a complete understanding of diagnoses among co-morbid patients, but we used validated algorithms to determine specific diagnoses, where applicable. Finally, we only report data from three Canadian provinces, and we do not know the extent to which these results are generalisable across Canada given marked differences in funding and organisation of services. Nevertheless, we were able to develop comparable indicators to examine data from three provinces, allowing us to provide an overview of changes in demographic and practice characteristics of psychiatrists over a 10-year period.

To conclude, this analysis shows a modest change in psychiatric practice characteristics despite changing psychiatric demographics during the past decade. While three provinces demonstrated similar overall trends, some differences between these three provinces underscore the importance of conducting pan-Canadian analyses to highlight particularities in workforce patterns.

## Disclaimers

### Data Access

The data that support the findings of this study reapproved for use by data stewards and accessed through a process managed by each province's data center. Requests to access the datasets using this study can be made to PopData (https://www.popdata.bc.ca/data_access), ICES (www.ices.on.ca/DAS; email: das@ices.on.ca) and Manitoba Centre for Health Policy (www.umanitoba.ca/manitoba­centre­for­health­policy/). We are not permitted to share the data used in this analysis with other researchers. The full dataset creation plan and underlying analytic code are available from the authors upon request, understanding that computer programs may rely upon coding templates or macros that are unique to this project and to individual data and are therefore either inaccessible or may require modification.

This study received in-kind coordination support from the Health Data Research Network (HDRN) Canada's Data Access Support Hub, in facilitating administrative data access across regions. HDRN Canada is supported by the Canadian Institutes of Health Research under Canada's Strategy for Patient-Oriented Research.

*Manitoba Centre for Health Policy*: The authors acknowledge the Manitoba Centre for Health Policy for use of data contained in the Manitoba Population Research Data Repository under project number: 2022-2020 (PHRPC No. P2022--01). The results and conclusions are those of the authors, and no official endorsement by the Manitoba Centre for Health Policy, Manitoba Health, or other data providers is intended or should be inferred. Data used in this study are from the Manitoba Population Research Data Repository housed at the Manitoba Centre for Health Policy, University of Manitoba and were derived from data provided by Manitoba Health.

*ICES*: This study was supported by ICES, which is funded by an annual grant from the Ontario Ministry of Health (MOH) and the Ministry of Long-Term Care (MLTC). This document used data adopted from the Statistics Canada Postal Code^OM^ Conversion File, which is based on data licensed from Canada Post Corporation, and/or data adapted from the Ontario Ministry of Health Postal Code Conversion File, which contains data copied under license from ©Canada Post Corporation and Statistics Canada. Parts of this material are based on data and/or information compiled and provided by MOH, CIHI and Statistics Canada. The analysis, conclusions, opinions and statements expressed herein are solely those of the authors and do not reflect those of the data sources; no endorsement is intended or should be inferred.

*Population Data BC*: Access to data provided by the Data Stewards is subject to approval, but can be requested for research projects through the Data Stewards or their designated service providers. The following data sets were used in this study: Consolidation file, Medical Services Plan, College of Physicians and Surgeons of BC,^23^ Vital Events Deaths, Discharge Abstract Database and National Ambulatory Care Reporting System. You can find further information regarding these data sets by visiting the PopData project webpage at: https://my.popdata.bc.ca/project_listings/22-068/collection_approval_dates. All inferences, opinions and conclusions drawn in this publication are those of the author(s) and do not reflect the opinions or policies of the Data Steward(s).

## Supplemental Material

sj-docx-1-cpa-10.1177_07067437251359183 - Supplemental material for Analysis of Demographic and Practice Characteristics of Psychiatrists in Three Canadian Provinces: Analyse des caractéristiques démographiques et de la pratique des psychiatres dans trois provinces canadiennes Supplemental material, sj-docx-1-cpa-10.1177_07067437251359183 for Analysis of Demographic and Practice Characteristics of Psychiatrists in Three Canadian Provinces: Analyse des caractéristiques démographiques et de la pratique des psychiatres dans trois provinces canadiennes by François Gallant, Ridhwana Kaoser, Sandra Peterson, Matt Dahl, Alison L. Park, James M. Bolton, Myriam Juda, Alan Katz, Jason Morrison, Benoit H. Mulsant, Philip G. Tibbo, Juveria Zaheer, Paul Kurdyak, M. Ruth Lavergne and David Rudoler in The Canadian Journal of Psychiatry
