# Supplementary material for: Analysis of Demographic and Practice Characteristics of Psychiatrists in Three Canadian Provinces: Analyse des caractéristiques démographiques et de la pratique des psychiatres dans trois provinces canadiennes
Source: Can J Psychiatry. 2025 Jul 21;71(2):129–38. doi: 10.1177/07067437251359183 (PMC12279761; doi:10.1177/07067437251359183)
Supplement: sj-docx-1-cpa-10.1177_07067437251359183 - Supplemental material for Analysis of Demographic and Practice Characteristics of Psychiatrists in Three Canadian Provinces: Analyse des caractéristiques démographiques et de la pratique des psychiatres dans trois provinces canadiennes [file sj-docx-1-cpa-10.1177_07067437251359183.docx]

# Supplementary Data

Access to data provided by the Data Steward(s) is subject to approval, but can be requested for research projects through the Data Steward(s) or their designated service providers. All inferences, opinions, and conclusions drawn in this publication are those of the author(s), and do not reflect the opinions or policies of the Data Steward(s).

Table A1: Description of Datasets

| Dataset | BC | MB | ON |
| --- | --- | --- | --- |
| Physician registry | College of Physicians and Surgeons of British Columbia: Provides data on all registered physicians. | Provider Registry (Physician Master File): Captures provider details such as specialty, date of birth/age, training location, years of practice, accepted payment methods, workload, and practice groups/practice location. The file is updated quarterly. The Electronic User Site Location (EUSL) data is used to determine practice location. | Corporate Provider Database: Holds details of all physicians and some non-physician providers (like chiropractors, physiotherapists, and optometrists) funded by the Ministry of Health through OHIP or other funding. It covers demographics, eligibility, specialty, and practice locations. The ICES Physician Database: Holds annual data on all Ontario physicians. It includes details from the Ontario Health Insurance Plan (OHIP), Corporate Provider Database (CPDB), Ontario Physician Human Resource Data Centre (OPHRDC), and the OHIP physician billing database. |
| Physician billings | The Medical Services Plan (MSP): Collects data on medically necessary services offered by fee-for-service practitioners to individuals covered by BC’s universal insurance program. The data also contains encounter (shadow) claims if submitted by alternatively-paid practitioners. | Medical Claims/Medical Services Data: Health data from Manitoba Health includes claims for physician visits, tests in offices and hospitals, payments for on-call agreements (e.g. anesthesiologists) not linked to specific patients, and details on physician specialties. | Ontario Health Insurance Plan: ICES received claims data mostly from the Ontario Health Insurance Plan, covering all healthcare providers eligible to claim under OHIP, such as physicians, groups, laboratories, and out-of-province providers. |
| Hospital separations | The CIHI Discharge Abstract Database: Contains information on discharges, transfers, and deaths of inpatients and day surgery patients from acute care hospitals, including inpatient acute, chronic, and rehabilitation cases. | The CIHI Discharge Abstract Database: Contains information on discharges, transfers, and deaths of inpatients and day surgery patients from acute care hospitals, including inpatient acute, chronic, and rehabilitation cases. | The CIHI Discharge Abstract Database: Contains information on discharges, transfers, and deaths of inpatients and day surgery patients from acute care hospitals, including inpatient acute, chronic, and rehabilitation cases. Ontario Mental Health Reporting System (OMHRS): Provides information on all individuals who received adult mental health services in Ontario, which includes location of admission, discharge info, mental and physical health. |
| Emergency department visits | The CIHI National Ambulatory Care Reporting System: Covers all levels of ambulatory care in Canada, including emergency departments, day surgery, medical and surgical day clinics in hospitals, communities, and private clinics. In BC, NACRS data is used to capture Emergency Department visits from reporting facilities ( 30 EDs in 2021-22). The combination of 2 data sources, NACRS and Medical Services Plan (MSP), contains the majority of ED visits in BC. | The Emergency Department Information System: Stores data about patients in a hospital’s emergency department. It includes patient demographics, arrival details, initial assessments like chief complaint and vital signs, CTAS score, healthcare providers, treatment orders, consultations, discharge diagnosis, and status. | The CIHI National Ambulatory Care Reporting System: Covers all levels of ambulatory care in Canada, including emergency departments, day surgery, medical and surgical day clinics in hospitals, communities, and private clinics. |
| Consolidation file | The Central Demographic File (formerly known as Consolidation File): Population Data BC’s central demographics file for research requests. It contains basic demographics such as age and sex, geo-codes indicating location of residence, and registration data. | Manitoba Health Insurance Registry: The registry includes individual-level demographics, family composition information, residential postal codes, and data fields for registration, birth, entry into province, and migration in/out of province. Data contains all individuals who have been registered with Manitoba Health. | Registered Persons Database: Provides basic demographic information about anyone who has ever received an Ontario health card number. Data supplied by the Ministry of Health is enriched with information from other ICES datasets. |

Table A2: Description of Variables

| **Variable** | **Definition** | **BC** | **MB** | **ON** |
| --- | --- | --- | --- | --- |
| Psychiatrist | Physicians with who made one or more claims as a psychiatrist between 2012/2013 and 2021/2022 | n/a | n/a | n/a |
| Age | December 31 of first calendar year within fiscal year - birth date | n/a | n/a | n/a |
| Sex/gender | Sex/gender | n/a | n/a | n/a |
| Years since Graduation | Years since MD awarded = Current year - grad year | n/a | n/a | n/a |
| Full-time status | Psychiatrists that fall in >30th percentile in billings per fiscal year | n/a | n/a | n/a |
| Rural/urban location (SACTYPE) | For each physician, the total number of patient contacts for each of the categorized SACTYPE areas (1, 2-3, 4-7) will be calculated. The category associated with the plurality of a physician's practice will be assigned. Use 2016 Census CSD to SACtype groupings.   1 = Metropolitan area (SACtype 1) 2 = Other urban area (SACtype 2-3) 3 = Rural area (SACtype 4-7) | n/a | n/a | n/a |
| Total visits | Number of visits provided by psychiatrist within fiscal year (unique combinations of physician, patient, and date) | Outpatient: service date not within DAD hospitalization.   Inpatient: service date during the period of DAD hospitalization | Outpatient: service date not within DAD hospitalization.   Inpatient: service date during the period of DAD or OMHRS hospitalization | Outpatient: Service date not withi DAD hospitalization   Inpatient: Any inpatient service date during the period of DAD hospitalization |
| Number of inpatient visits | Number of inpatient visits provided by each psychiatrist within fiscal year (unique combinations of physician, patient, date, where patient was in hospital according to DAD | Inpatient: service date during the period of DAD hospitalization | Inpatient: Service date during the period of DAD or OMHRS hospitalization | Inpatient: Any inpatient service date during the period of DAD hospitalization |
| Number of outpatient visits | Number of outpatient visits provided by each psychiatrist within fiscal year (unique combinations of physician, patient, date, where patient was not in hospital according to DAD) | Outpatient: service date not within DAD hospitalization. | Outpatient: Service date not within DAD or OMHRS hospitalization | Outpatient: Service date not within DAD hospitalization |
| Total individuals seen | Total number of patients (unique StudyIDs) seen by each psychiatrist (in or out of hospital). |  |  |  |
| Total outpatients | Total number of outpatients (unique combinations of physician, patient, date, where patient was not in hospital according to DAD) | Outpatient: service date not within DAD hospitalization. | Outpatient: Service date not within DAD or OMHRS hospitalization | Outpatient: Service date not within DAD hospitalization |
| Number of new outpatients | Number of new patients (not seen in the previous two years in any setting in Ontario and Manitoba; and not seen in the previous 12 months in British Columbia) seen by each psychiatrist at a time when the patient was not in hospital according to DAD. For this variable the index date is the patient's first outpatient visit with each psychiatrist within each FY and look-back is the 2 years prior to that date (BC-1 year). | Outpatient: service date not within DAD hospitalization. | Outpatient: Service date not within DAD or OMHRS hospitalization | Outpatient: Service date not within DAD hospitalization |
| Continuity of Care - 1 to 2 visits | Number of unique patients seen by the same psychiatrist for 1 to 2 outpatient visits within the current fiscal year | Outpatient: service date not within DAD hospitalization. | Outpatient: Service date not within DAD or OMHRS hospitalization | Outpatient: Service date not within DAD hospitalization |
| Continuity of Care - 3 to 5 visits | Number of unique patients seen by the same psychiatrist for 3 to 5 outpatient visits within the current fiscal year | Outpatient: service date not within DAD hospitalization. | Outpatient: Service date not within DAD or OMHRS hospitalization | Outpatient: Service date not within DAD hospitalization |
| Continuity of Care - 6 to 11 visits | Number of unique patients seen by the same psychiatrist for 6 to 11 outpatient visits within the current fiscal year | Outpatient: service date not within DAD hospitalization. | Outpatient: Service date not within DAD or OMHRS hospitalization | Outpatient: Service date not within DAD hospitalization |
| Continuity of Care - 12 or more visits | Number of unique patients seen by the same psychiatrist for 12 or more outpatient visits within the current fiscal year | Outpatient: service date not within DAD hospitalization. | Outpatient: Service date not within DAD or OMHRS hospitalization | Outpatient: Service date not within DAD hospitalization |
| Number of days with patient contacts | Total number of calendar days on which one or more patient contacts occurred |  |  |  |
| Saw < 100 patients/year | Saw < 100 patients (unique StudyIDs, seen in or out of hospital). |  |  |  |
| Total psychotherapy visits | Sum of inpatient and outpatient psychotherapy visits | Billing code 00630, 00631, 00632, 00633, 00635, 00636, 00638, 00639, 00663-00681, 60630, 60631, 60632, 60633, 60635, 60636, 60638, 60639 | Billing code K197, K195, K208, K203, K204, K205, K206, K194 OR K199 | billing codes 8581, 8444, 8446, 8533, 8585 |
| Number of virtual care (outpatient only) | Unique psychiatrist/patient/date that include billing of relevant fee item for phone or video visits, where the patient was not in hospital according to DAD | Billing codes 60610, 60613, 60622, 60625, 60614, 60626, 60607, 60608, 60630, 60631, 60632, 60633, 60635, 60636, 60638, 60639 | Billing codes (from November 2019 only): B099A, B100A, B200A, B101A, B201A, B102A, B202A, B103A, B203A, K082 | Billing codes 8533, 8786, 8535, 8321, 8527 |
| Number of mental health visits | Unique combinations of physician/patient/date that include ICD codes that correspond to mental health contacts but do not include ICD codes that correspond to substance use disorders | "295 Schizophrenia 296 Manic-depressive psychoses, involutional melancholia 297 Other paranoid states 298 Other psychoses  Non-Psychotic Disorders 300 Anxiety neurosis, hysteria, neurasthenia, obsessive-compulsive neurosis, reactive depression 301 Personality disorders 302 Sexual deviations 306 Psychosomatic illness 307 Special symptoms or syndromes not elsewhere classified 308 Acute reaction to stress 309 Adjustment disorder with depressed mood 311 Depressive disorder 314 Hyperkinetic syndrome of childhood  (also BC only codes:  50B Anxiety/depression | "295 Schizophrenia 296 Manic-depressive psychoses, involutional melancholia 297 Other paranoid states 298 Other psychoses  Non-Psychotic Disorders 300 Anxiety neurosis, hysteria, neurasthenia, obsessive-compulsive neurosis, reactive depression 301 Personality disorders 302 Sexual deviations 306 Psychosomatic illness 307.1 Anorexia Nervosa 307.5 Other and unspecified disorders of eating 308 Acute reaction to stress 309 Adjustment disorder with depressed mood 311 Depressive disorder 314 Hyperkinetic syndrome of childhood | "295 Schizophrenia 296 Manic-depressive psychoses, involutional melancholia 297 Other paranoid states 298 Other psychoses  Non-Psychotic Disorders 300 Anxiety neurosis, hysteria, neurasthenia, obsessive-compulsive neurosis, reactive depression 301 Personality disorders 302 Sexual deviations 306 Psychosomatic illness 307.1 Anorexia Nervosa 307.5 Other and unspecified disorders of eating 308 Acute reaction to stress 309 Adjustment disorder with depressed mood 311 Depressive disorder 314 Hyperkinetic syndrome of childhood |
| Number of substance use visits | Unique combinations of physician/patient/date that include ICD codes that correspond to substance use disorders | Substance Use Disorders 303 Alcoholism 304 Drug dependence | Substance Use Disorders 303 Alcoholism 304 Drug dependence | Substance Use Disorders 303 Alcoholism 304 Drug dependence |
| Number of non-mental health/non-substance use visits | Unique combinations of physician/patient/date that DO NOT include ICD codes that correspond to mental or substance use disorders | All other codes | All other codes | All other codes |
| Number of visits for patients treated for substance use | Unique combinations of physician/patient/date for patients treated (within same fiscal year) for substance use in physician, ED or hospital data | ICD9: 303, 304 DX10CODE1-NACRS: F10-19, F55, F63.0 DX10CODE1: F10-F19, F55, F63.0 | ICD9: 303, 304 DX10CODE1-NACRS: F10-19, F55, F63.0 DX10CODE1: F10-F19, F55, F63.0  OMHRS: 291.x (all 291 codes, excluding 291.82), 292.x (all 292 codes, excluding 292.85), 303.x (all 303 codes), 304.x (all 304 codes), 305.x (all 305 codes), 312.31. Provisional=4 | ICD9: 303, 304 EDIS/ICD-10: F10-19, F55, F63.0 ICD10: F10-F19, F55, F63.0 |
| Number of visits for patients treated for schizophrenia, schizoaffective, and psychotic disorders NOS | Number of visits with individuals flagged as having schizophrenia, schizoaffective disorder or psychotic disorders NOS using the algorithm as described for person_FY. Count the number of visits for people flagged for the same FY in the person_FY data (recall, that flag used look-back of 5 years plus the current FY). | ICD9: 295 and 298 DX10CODE1: F20, F25, and F29 | ICD9: 295 and 298 DX10CODE1: F20, F25, and F29 OMHRS DSM-IV schizophrenia (295), schizoaffective disorder (295), and psychotic disorders (298) | ICD9: 295 and 298 DX10CODE1: F20, F25, and F29 |

| Table A3. Personal characteristics of psychiatrists practicing in British Columbia from 2012/13 to 2021/22 | | | | | | | | | |  |
| --- | --- | --- | --- | --- | --- | --- | --- | --- | --- | --- |
| FY | 2012/13 | 2013/14 | 2014/15 | 2015/16 | 2016/17 | 2017/18 | 2018/19 | 2019/2020 | 2020/21 | 2021/2022 |
| n | 754 | 782 | 810 | 832 | 830 | 825 | 829 | 841 | 855 | 870 |
| Age (years), mean (SD) | 52.48 (11.68) | 52.60 (11.53) | 52.32 (11.70) | 52.34 (11.87) | 52.24 (11.88) | 52.31 (11.81) | 52.22 (11.75) | 52.07 (11.70) | 51.81 (11.68) | 51.75 (11.77) |
| Median (IQR) | 52.00  (43.00-61.00) | 52.00  (43.00-61.00) | 51.00  (43.00-61.00) | 51.00  (43.00-61.00) | 51.00  (43.00-61.00) | 51.00  (43.00-61.00) | 51.00  (43.00-61.00) | 51.00  (43.00-61.00) | 51.00  (43.00-60.00) | 51.00  (43.00-60.00) |
| Sex/gender, n (%) female | 295 (39.1) | 310 (39.6) | 335 (41.4) | 352 (42.3) | 353 (42.5) | 355 (43.0) | 361 (43.5) | 370 (44.0) | 380 (44.4) | 392 (45.1) |
| Years since MD grad mean (SD) | 25.65 (12.29) | 25.73 (12.18) | 25.34 (12.37) | 25.38 (12.55) | 25.25 (12.53) | 25.26 (12.47) | 25.23 (12.39) | 24.99 (12.29) | 24.67 (12.21) | 24.61 (12.25) |
| Median (IQR) | 25.00  (16.00-34.00) | 25.00  (16.00-35.00) | 24.00  (16.00-34.00) | 24.00  (16.00-34.00) | 24.00  (16.00-34.00) | 23.00  (16.00-34.00) | 23.00  (16.00-34.00) | 23.00  (15.00-34.00) | 23.00  (15.00-34.00) | 24.00  (14.00-34.00) |
| Full-time, n (%) | 527 (69.9) | 547 (69.9) | 567 (70.0) | 582 (70.0) | 581 (70.0) | 577 (69.9) | 580 (70.0) | 588 (69.9) | 598 (69.9) | 609 (70.0) |
| Metropolitan area, n (%) | 610 (80.9) | 633 (81.0) | 653 (80.7) | 676 (81.3) | 669 (80.8) | 662 (80.3) | 661 (79.8) | 672 (80.0) | 676 (79.2) | 690 (79.5) |
| Other urban area, n (%) | 124 (16.5) | 124 (16.5) | 125 (16.0) | 133 (16.4) | 131 (15.8) | 135 (16.3) | 140 (17.0) | 142 (17.2) | 144 (17.1) | 151 (17.4) |
| Rural area, n (%) | 20 (2.7) | 20 (2.7) | 23 (2.9) | 23 (2.8) | 25 (3.0) | 24 (2.9) | 22 (2.7) | 25 (3.0) | 24 (2.9) | 27 (3.1) |

| Table A4. Personal characteristics of psychiatrists practicing in Manitoba from 2012/13 to 2021/22 | | | | | | | | | |  |
| --- | --- | --- | --- | --- | --- | --- | --- | --- | --- | --- |
| FY | 2012/13 | 2013/14 | 2014/15 | 2015/16 | 2016/17 | 2017/18 | 2018/19 | 2019/2020 | 2020/21 | 2021/2022 |
| n | 155 | 159 | 164 | 168 | 184 | 189 | 190 | 188 | 191 | 186 |
| Age (years), mean (SD) | 52.97 (11.36) | 53.38 (11.67) | 53.24 (12.60) | 53.73 (12.21) | 53.14 (12.52) | 52.69 (12.31) | 52.74 (12.63) | 52.02 (12.89) | 52.08 (12.75) | 52.19 (13.06) |
| Median (IQR) | 53.50  (44.00-61.00) | 54.00  (45.00-61.00) | 54.00  (43.00-61.00) | 55.00  (43.00-62.00) | 54.00  (43.00-62.00) | 51.00  (42.50-61.00) | 52.00  (42.00-62.00) | 51.00  (41.00-61.50) | 50.00  (42.00-62.00) | 50.00  (41.00-62.00) |
| Sex/gender, n (%) female | 50 (32.89) | 54 (34.39) | 58 (35.80) | 56 (33.73) | 59 (32.24) | 59 (31.38) | 61 (32.28) | 61 (32.45) | 61 (32.11) | 59 (31.72) |
| Years since MD grad, mean (SD) | 25.95 (12.04) | 26.39 (12.34) | 26.42 (12.56) | 26.64 (12.86) | 26.13 (13.10) | 25.63 (12.91) | 25.49 (13.22) | 25.75 (12.88) | 26.64 (12.54) | 27.50 (12.53) |
| Median (IQR) | 27.00  (16.50-33.00) | 28.00  (16.00-34.00) | 27.50  (16.00-34.00) | 28.00  (16.00-35.00) | 25.00  (15.00-35.00) | 25.00  (15.00-34.50) | 24.00  (15.00-35.00) | 24.00  (15.00-36.00) | 25.00  (16.00-36.00) | 26.00  (17.00-37.00) |
| Full-time, n (%) | 108 (69.68) | 111 (69.81) | 114 (69.51) | 117 (69.64) | 128 (69.57) | 132 (69.84) | 133 (70.00) | 132 (69.84) | 133 (69.63) | 130 (69.89) |
| Metropolitan area, n (%) | 134 (86.5) | 142 (89.3) | 143 (87.2) | 147 (87.5) | 161 (87.5) | 166 (87.8) | 162 (85.3) | 162 (86.2) | 165 (86.4) | 160 (86.0) |
| Other urban area, n (%) | 13 (8.4) | 11 (6.9) | 11 (6.7) | 14 (8.3) | 16 (8.7) | 17 (9.0) | 20 (10.5) | 17 (9.0) | 15 (7.9) | 13 (7.0) |
| Rural area, n (%) | 6 (3.9) | 6 (3.8) | 10 (6.1) | 7 (4.2) | 7 (3.8) | 6 (3.2) | 8 (4.2) | 9 (4.8) | 11 (5.8) | 13 (7.0) |

| Table A5. Personal characteristics of psychiatrists practicing in Ontario from 2012/13 to 2021/22 | | | | | | | | | |  |
| --- | --- | --- | --- | --- | --- | --- | --- | --- | --- | --- |
| FY | 2012/13 | 2013/14 | 2014/15 | 2015/16 | 2016/17 | 2017/18 | 2018/19 | 2019/2020 | 2020/21 | 2021/2022 |
| n | 2092 | 2131 | 2163 | 2187 | 2207 | 2229 | 2237 | 2282 | 2291 | 2339 |
| Age (years), mean (SD) | 55.33 (13.43) | 55.18 (13.43) | 55.10 (13.51) | 55.07 (13.34) | 54.96 (13.27) | 54.81 (13.46) | 54.54 (13.64) | 54.25 (13.80) | 53.80 (13.92) | 53.58 (13.99) |
| Median (IQR) | 56.00  (45.00-64.00) | 56.00  (44.00-64.00) | 56.00  (44.00-64.00) | 56.00  (44.00-65.00) | 56.00  (44.00-65.00) | 55.00  (43.00-65.00) | 55.00  (43.00-65.00) | 54.00  (43.00-65.00) | 53.00  (42.00-65.00) | 52.00  (42.00-65.00) |
| Sex/gender, n (%) female | 814 (38.91) | 849 (39.84) | 879 (40.64) | 902 (41.24) | 931 (42.18) | 957 (42.95) | 992 (44.35) | 1031 (45.18) | 1056 (46.09) | 1095 (46.81) |
| Years since MD grad, mean (SD) | 28.70 (13.29) | 28.57 (13.48) | 28.49 (13.58) | 28.58 (13.79) | 28.45 (13.96) | 28.33 (14.09) | 27.99 (14.26) | 27.59 (14.42) | 27.04 (14.50) | 27.76 (14.26) |
| Median (IQR) | 29.00 (18.00-38.00) | 29.00  (17.00-38.00) | 29.00  (17.00-38.00) | 29.00  (17.00-39.00) | 29.00  (16.00-39.00) | 29.00  (16.00-39.00) | 28.00  (15.00-39.00) | 26.00  (15.00-39.00) | 25.00  (14.00-38.00) | 26.00  (15.00-39.00) |
| Full-time, n (%) | 1464 (69.98) | 1491 (69.97) | 1514 (70.00) | 1530 (69.96) | 1544 (69.96) | 1559 (69.97) | 1565 (69.96) | 1597 (69.98) | 1603 (69.97) | 1637 (69.99) |
| Metropolitan area, n (%) | 1950 (90.5) | 1982 (93.1) | 2013 (93.2) | 2046 (93.6) | 2061 (93.5) | 2073 (93.2) | 2082 (93.1) | 2133 (93.6) | 2144 (93.6) | 2173 (93.1) |
| Other urban area, n (%) | 90 (4.3) | 94 (4.4) | 94 (4.4) | 89 (4.1) | 99 (4.5) | 103 (4.6) | 105 (4.7) | 97 (4.3) | 102 (4.5) | 103 (4.4) |
| Rural area, n (%) | 45 (2.2) | 52 (2.4) | 52 (2.4) | 50 (2.3) | 45 (2.0) | 48 (2.2) | 49 (2.2) | 50 (2.2) | 44 (1.9) | 59 (2.5) |

| Table A6. Practice characteristics of psychiatrists in British Columbia from 2012/13 to 2020/21. | | | | | | | | | | | |  |
| --- | --- | --- | --- | --- | --- | --- | --- | --- | --- | --- | --- | --- |
| FY |  | 2012/13 | 2013/14 | 2014/15 | 2015/16 | 2016/17 | 2017/18 | 2018/19 | 2019/20 | 2020/21 | 2021/22 | |
| Total # of visits | Mean (SD) | 1333.9 (1361.1) | 1352.86 (1387.80) | 1350.04 (1312.46) | 1353.64 (1280.63) | 1389.31 (1309.97) | 1355.84 (1243.57) | 1373.44 (1248.34) | 1359.05 (1274.18) | 1474.81 (1421.52) | 1436.8 (1505.7) | |
|  | Median (IQR) | 1076.5  (517-1740) | 1087  (522-1761) | 1072.50  (553-1752) | 1124  (556-1792) | 1123  (585-1839) | 1105  (576-1824) | 1101  (567-1879) | 1081  (548-1860) | 1205  (619-1975) | 1137  (588-1911) | |
| Total # of inpatient visits | Mean (SD) | 389.9 (625.0) | 400.45 (636.70) | 398.51 (601.30) | 398.68 (592.41) | 413.22 (613.36) | 414.05 (597.71) | 421.47 (638.92) | 418.75 (631.03) | 396.98 (624.86) | 409.7 (640.8) | |
|  | Median (IQR) | 45  (2-640) | 41.50  (2-641) | 54.50  (3-687) | 58  (3-703.5) | 56  (2-711) | 67  (2-722) | 71  (3-702) | 69  (3-682) | 51  (4-642) | 53.5  (4-690) | |
| Total # of outpatient visits | Mean (SD) | 944.0 (1155.7) | 952.41 (1185.24) | 951.53 (1141.85) | 954.97 (1117.87) | 976.09 (1145.36) | 941.78 (1109.25) | 951.98 (1091.74) | 940.30 (1115.42) | 1077.83 (1281.81) | 1027.2 (1369.1) | |
|  | Median (IQR) | 737.5  (308-1280) | 710  (311-1289) | 717.50  (325-1264) | 708  (309.50-1250) | 737  (341-1276) | 726  (312-1243) | 725  (318-1247) | 691  (305-1222) | 782  (330-1418) | 718.5  (332-1290) | |
| Total # individuals seen | Mean (SD) | 273.5 (304.7) | 282.40 (322.29) | 282.05 (297.38) | 288 (315.69) | 296.61 (306.45) | 299.57 (297.73) | 305.19 (306.01) | 304.58 (311.13) | 303.35 (300.94) | 313.4 (327.2) | |
|  | Median (IQR) | 202.5  (103-365) | 208  (105-375) | 208  (103-370) | 203.50  (100-380) | 220.50  (108-403) | 223  (111-410) | 228  (115-408) | 225  (115-401) | 222  (97-408) | 225.5  (103-422) | |
| # of new outpatients (not seen in prior 12m) | Mean (SD) | 117.0 (212.0) | 122.49 (231.34) | 119.62 (201.68) | 122.67 (218.26) | 126.32 (199.78) | 127.22 (193.75) | 131.76 (202.45) | 131.20 (204.91) | 130.94 (181.43) | 133.3 (197.4) | |
|  | Median (IQR) | 76  (35-151) | 79.50  (33-157) | 79.50  (32-154) | 80  (33-153) | 84  (35-161) | 85  (32-168) | 85  (33-165) | 86  (31-163) | 79  (26-165) | 80  (26-172) | |
| % patients seen for 1-2 outpatient visits | Mean (SD) | 48.6  (23.8) | 49.40 (24.02) | 49.30 (24.67) | 49.65 (24.38) | 49.65 (24.67) | 51.43 (25.69) | 51.77  (25.42) | 51.76 (25.35) | 47.75 (26.25) | 50.0 (26.8) | |
|  | Median (IQR) | 47.2  (30.8-63.3) | 47.53  (31.82-64.96) | 46.15  (31.40-65.15) | 47.83  (31.25-65.77) | 48.83  (31.15-66.53) | 50  (30.86-70.93) | 49.74  (32.25-69.23) | 50.65  (32.37-68.95) | 46.54  (26.27-66.57) | 49.3  (28.6-69.1) | |
| % patients seen for 3-5 outpatient visits | Mean (SD) | 22.4  (11.0) | 22.38 (11.56) | 22.38 (11.79) | 22.77 (11.75) | 22.78 (11.94) | 22.35 (12.70) | 22.21 (12.26) | 22.80 (12.43) | 21.46 (12.71) | 21.6  (13.2) | |
|  | Median (IQR) | 23.1  (14.8-29.2) | 22.73  (15-30.26) | 23.08  (14.69-29.82) | 23.28  (15.67-30.43) | 23.53  (14.88-30.20) | 22.19  (14.04-30.43) | 22.49  (14.69-30.25) | 23.29  (13.87-31.58) | 21.24  (12.57-29.90) | 22.2  (12.0-29.6) | |
| % patients seen for 6-11 outpatient visits | Median (IQR) | 16.9  (11.4) | 16.48 (11.18) | 16.87 (12.06) | 16.37 (11.51) | 16.61 (12.32) | 15.73 (12.01) | 15.58 (12.32) | 15.41 (12.68) | 17.36 (13.23) | 16.0  (13.2) | |
|  | Mean (SD) | 15.8  (8.1-24.6) | 15.79  (7.92-23.81) | 16.28  (7.76-24.07) | 15.99  (7.35-23.86) | 15.90  (7.25-23.68) | 13.71  (6.09-23.81) | 13.68  (5.74-23.06) | 12.69  (5.01-23.20) | 16.09  (7.03-24.86) | 13.1  (5.3-24.7) | |
| % patients seen for 12+ outpatient visits | Median (IQR) | 12.1  (17.5) | 11.74 (16.86) | 11.45 (16.73) | 11.21 (17.32) | 10.97 (16.90) | 10.49 (16.99) | 10.45  (16.86) | 10.02 (17.14) | 13.42 (19.98) | 12.5  (20.2) | |
|  | Mean (SD) | 4.7  (1.1-15.1) | 4.26  (0.91-15.72) | 4.35  (0.75-14.96) | 3.70  (0.46-13.87) | 4.14  (0.49-12.90) | 3.16  (0.23-12.50) | 2.96  (0.36-12.23) | 2.50  (0.03-10.43) | 4.93  (0.79-16.87) | 3.5  (0.3-15.2) | |
| # billing days | Mean (SD) | 168.2  (68.5) | 167.87 (69.34) | 168.34 (68.55) | 168.93 (70.22) | 172.55 (68.98) | 169.85 (66.74) | 170.20  (67.55) | 169.02 (69.95) | 182.53 (76.55) | 174.3  (74.8) | |
|  | Median (IQR) | 181.5  (125-218) | 182.50  (118-221) | 183.50  (124-220) | 184  (126.50-222) | 187  (130-224) | 183  (129-221) | 183  (129-221) | 181  (125-221) | 199  (139-237) | 190  (130-227) | |
| Saw <100 patients/year | (n %) | 180 (23.9) | 190 (24.3) | 198 (24.4) | 203 (24.4) | 181 (21.8) | 183 (22.2) | 171 (20.6) | 186 (22.1) | 218 (25.5) | 209 (24.0) | |

| Table A7. Practice characteristics of psychiatrists in Manitoba from 2012/13 to 2020/21. | | | | | | | | | | | |  |
| --- | --- | --- | --- | --- | --- | --- | --- | --- | --- | --- | --- | --- |
| FY |  | 2012/13 | 2013/14 | 2014/15 | 2015/16 | 2016/17 | 2017/18 | 2018/19 | 2019/20 | 2020/21 | 2021/22 | |
| Total # of visits | Mean (SD) | 1300.21 (1072.41) | 1257.14 (1073.30) | 1246.20 (1092.76) | 1223.80 (1059.94) | 1172.64 (1015.49) | 1166.77 (1043.67) | 1409.16 (1250.53) | 1493.71 (1356.52) | 1626.60 (1539.05) | 1602.89 (1585.82) | |
|  | Median (IQR) | 1047 (449-1941) | 894 (362-1999) | 972.50 (345-1941.50) | 1025 (360-1883) | 910.50 (398.50-1775) | 889 (326-1774) | 992.50 (421-2205) | 1157 (455-2244) | 1317 (489-2552) | 1109 (481-2499) | |
| Total # of inpatient visits | Mean (SD) | 337 (560.60) | 322.50 (579.34) | 311.29 (580.34) | 292.52 (533.59) | 283.11 (492.93) | 285.16 (510.25) | 486.61 (800.51) | 521.84 (901.93) | 517.74 (984.59) | 486.91 (917.11) | |
|  | Median (IQR) | 28 (1-581) | 20 (1-346) | 24.50 (0-274) | 20 (0-268) | 22 (0-314.50) | 14 (0-249) | 28.50 (1-656) | 34 (1-601) | 23 (1-701) | 32.50 (2-789) | |
| Total # of outpatient visits | Mean (SD) | 963.21 (877.16) | 934.65 (889.83) | 934.91 (889.33) | 931.28 (888.22) | 889.53 (848.45) | 881.61 (863.31) | 922.56 (856.53) | 971.87 (883.61) | 1108.85 (1009.70) | 1115.98 (1007.61) | |
|  | Median (IQR) | 756 (280-1410) | 658 (236-1435) | 681.50 (275.50-1376.50) | 704 (269.50-1455) | 673.50 (214.50-1340) | 662 (207-1302) | 715 (301-1284) | 705 (338-1408) | 810 (361-1607) | 834 (377-1529) | |
| Total # individuals seen | Mean (SD) | 241.46 (189.92) | 231.45 (191.50) | 231.10 (188.94) | 230.25 (185.42) | 231.59 (189.75) | 231.91 (194.42) | 250.09 (198.13) | 257.02 (196.69) | 245.57 (195.01) | 257.17 (205.17) | |
|  | Median (IQR) | 222 (70-373) | 208 (59-345) | 199.50 (68-357.50) | 209.50 (67-341.50) | 207.50 (82-324) | 197 (77-332) | 206.50 (91-375) | 237 (100-375) | 210 (94-338) | 218.50 (93-386) | |
| # of new outpatients (not seen in prior 12m) | Mean (SD) | 85.05 (78.29) | 82.88 (80.62) | 85.59 (81.09) | 87.73 (83.38) | 95.27 (91.98) | 94.13 (92.64) | 107.98 (112.11) | 108.08 (109.55) | 95.24 (105.15) | 106.58 (111.31) | |
|  | Median (IQR) | 63 (21-129) | 60 (19-119) | 59.50 (19-133) | 61.50 (23-131) | 67.50 (23-154) | 68 (18-135) | 73 (23-151) | 76 (28-150) | 70 (24-131) | 81 (28-153) | |
| % patients seen for 1-2 outpatient visits | Mean (SD) | 53.21 (26.59) | 51.30 (27.27) | 51.55 (26.33) | 52.73 (27.43) | 56.12 (27.82) | 56.33 (28.24) | 55.67 (27.08) | 55.64 (26.35) | 53.08 (27.17) | 53.86 (27.26) | |
|  | Median (IQR) | 54.24 (32.34-72.87) | 54.64 (27.54-74) | 52.16 (31.60-70.37) | 53.16 (30.14-71.91) | 59.13 (33.36-75.43) | 56.35 (35.45-76.92) | 56.08 (33.11-75.79) | 54.43 (34.36-76.19) | 53.33 (30.85-75.76) | 54.62 (31.85-77.18) | |
| % patients seen for 3-5 outpatient visits | Mean (SD) | 16.29 (10.02) | 17.07 (11.54) | 17.88 (12) | 16.94 (13.02) | 15.53 (12) | 15.19 (9.93) | 15.86 (9.75) | 15.80 (9.64) | 14.19 (8.91) | 14.34 (9.25) | |
|  | Median (IQR) | 15.61 (10.47-23.08) | 16.04 (10.08-21.94) | 17.05 (11.11-24.29) | 16.28 (7.69-23.29) | 13.93 (7.63-22.24) | 15.64 (7.92-22.33) | 15.23 (8.79-22.92) | 15.25 (9.81-22.61) | 13.55 (8.24-19.74) | 13.54 (8.33-19.55) | |
| % patients seen for 6-11 outpatient visits | Median (IQR) | 14.60 (9.84) | 14.80 (9.99) | 14.76 (10.45) | 14.85 (11.78) | 13.64 (10.99) | 14.46 (12.18) | 14.78 (11.79) | 14.37 (11.15) | 14.31 (10.15) | 14.33 (10.14) | |
|  | Mean (SD) | 14.14 (8.05-20.83) | 14.29 (6.67-21.81) | 14.01 (5.94-21.84) | 13.18 (6.35-21.05) | 12.78 (4.94-20.04) | 12.90 (5.26-20.31) | 13.09 (5.63-21.55) | 13.06 (6.19-20.17) | 13.57 (6.25-20.09) | 14.04 (6.67-20.60) | |
| % patients seen for 12+ outpatient visits | Median (IQR) | 15.89 (18.93) | 16.83 (19.37) | 15.82 (18.27) | 15.48 (18.71) | 14.72 (18.29) | 14.02 (18.56) | 13.68 (18.32) | 14.19 (17.90) | 18.42 (20.39) | 17.47 (20.26) | |
|  | Mean (SD) | 8.39 (2.94-24.66) | 8.50 (3.45-25.95) | 8.48 (3.17-24.01) | 8.20 (1.61-23.33) | 7.26 (1.85-20.36) | 6.98 (1.12-19.48) | 7.29 (1.01-17.90) | 7.64 (1.90-17.71) | 11.44 (3.41-27) | 9.54 (3.51-24.09) | |
| # billing days | Mean (SD) | 185.39 (84.20) | 181.09 (84.74) | 181.51 (88.06) | 179.16 (89.08) | 174.39 (88.84) | 171.03 (93.97) | 194.72 (104.02) | 201.37 (101.66) | 215.77 (100.70) | 213.39 (100.30) | |
|  | Median (IQR) | 212 (118-246) | 208 (114-242) | 211 (120-244) | 207 (123-249) | 199.50 (105.50-243.50) | 203 (93-244) | 201.50 (119-268) | 214 (139-265) | 233 (147-289) | 228 (145-301) | |
| Saw <100 patients/year | (n %) | 49 (31.61) | 53 (33.33) | 58 (35.37) | 55 (32.74) | 54 (29.35) | 58 (30.69) | 52 (27.37) | 47 (24.87) | 50 (26.18) | 49 (26.34) | |

| Table A8. Practice characteristics of psychiatrists in Ontario from 2012/13 to 2020/21. | | | | | | | | | | | |  |
| --- | --- | --- | --- | --- | --- | --- | --- | --- | --- | --- | --- | --- |
| FY |  | 2012/13 | 2013/14 | 2014/15 | 2015/16 | 2016/17 | 2017/18 | 2018/19 | 2019/20 | 2020/21 | 2021/22 | |
| Total # of visits | Mean (SD) | 1362.13 (1275.58) | 1382.91 (1312.44) | 1380.67 (1311.64) | 1388.91 (1310.64) | 1377.98 (1313.87) | 1361.68 (1331.75) | 1366.96 (1304.77) | 1333.96 (1291.82) | 1394.08 (1333.88) | 1344.19 (1298.98) | |
|  | Median (IQR) | 1089.50 (502.50-1791.50) | 1092 (529-1826) | 1074 (515-1827) | 1083 (525-1821) | 1077 (501-1831) | 1058.50 (486-1765.50) | 1067 (515-1788) | 1039.50 (471-1738) | 1090 (487-1884) | 1049 (482-1763) | |
| Total # of inpatient visits | Mean (SD) | 311.55 (633.79) | 325.39 (642.32) | 327.79 (645.62) | 339.69 (660.97) | 344.54 (662.90) | 352.77 (681.79) | 364.20 (685.36) | 356.78 (675.34) | 321.17 (613.80) | 295.92 (549.17) | |
|  | Median (IQR) | 10 (0-237) | 14 (0-276) | 13 (0-304) | 13 (0-311) | 14 (0-321) | 17 (0-327.50) | 19 (1-348) | 21 (1-346) | 18 (1-314) | 17 (1-299) | |
| Total # of outpatient visits | Mean (SD) | 1050.59 (1057.73) | 1057.52 (1096.75) | 1052.88 (1087.43) | 1049.22 (1079.74) | 1033.45 (1087.77) | 1008.91 (1090.78) | 1002.76 (1067.52) | 977.17 (1060.11) | 1072.91 (1132.65) | 1048.27 (1121.23) | |
|  | Median (IQR) | 829.50 (341.50-1425) | 830 (337-1431) | 820 (341-1429) | 825 (336-1409) | 811 (336-1394) | 759 (303.50-1380.50) | 779 (326-1351) | 731.50 (310-1311) | 822 (330-1465) | 805 (344-1406) | |
| Total # individuals seen | Mean (SD) | 292.18 (360.37) | 299.26 (357.12) | 302.21 (360.46) | 311.93 (375.44) | 320.56 (397.95) | 328.32 (400.03) | 338.69 (387.62) | 332.83 (382.13) | 310.16 (350.71) | 324.37 (378.06) | |
|  | Median (IQR) | 185 (77-374) | 197 (79-393) | 197 (81-400) | 204 (85-407) | 205 (86-417) | 212 (90-420.50) | 225 (93-439) | 222 (92-433) | 199 (82-414) | 215 (86-435) | |
| # of new outpatients (not seen in prior 12m) | Mean (SD) | 115.69 (146.04) | 116.10 (146.67) | 115.95 (143.95) | 117.96 (145.66) | 117.58 (148.28) | 117.94 (149.61) | 119.26 (148.80) | 118.47 (153.17) | 115.41 (151.48) | 116.61 (153.34) | |
|  | Median (IQR) | 65 (26-146.50) | 66 (26-147) | 66 (26-149) | 67 (26-154) | 67 (26-154) | 66 (26-156) | 68 (26-155) | 67 (26-152) | 65 (25-147) | 69 (26-146) | |
| % patients seen for 1-2 outpatient visits | Mean (SD) | 48.21 (25.48) | 49.66 (25.93) | 49.96 (25.96) | 50.74 (26.03) | 51.65 (26.05) | 53.14 (26.47) | 54.01 (26.35) | 54.68 (26.27) | 50.44 (27.09) | 52.30 (27.11) | |
|  | Median (IQR) | 47.24 (27.79-66.34) | 48.91 (28.83-69.01) | 49.48 (29.36-69.02) | 50 (29.78-70.12) | 52.39 (30.61-70.93) | 53.49 (32.14-73.88) | 54.79 (33.57-75) | 56 (34.78 - 75.20) | 51.35 (28.57 - 71.11) | 54.58 (31.13-72.47) | |
| % patients seen for 3-5 outpatient visits | Mean (SD) | 20.62 (11.78) | 20.08 (11.91) | 20.04 (11.98) | 20.20 (12.03) | 20.10 (12.05) | 20.05 (12.27) | 19.88 (12.21) | 20.26 (12.67) | 18.94 (12.23) | 19.02 (11.95) | |
|  | Median (IQR) | 20.37 (12.38-27.61) | 19.74 (11.76-27.52) | 19.34 (12.11-26.98) | 19.72 (11.54-27.53) | 19.45 (11.69-27.51) | 19.44 (11.11-27.59) | 19.15 (11.11-27.40) | 19.57 (11.11 - 28.17) | 18.26 (9.92 - 25.94) | 18.37 (10.20-26.50) | |
| % patients seen for 6-11 outpatient visits | Median (IQR) | 15.90 (11.55) | 15.35 (11.44) | 15.55 (11.84) | 14.98 (11.34) | 14.68 (11.25) | 14.25 (11.41) | 14.14 (11.38) | 13.44 (10.92) | 15.02 (11.99) | 14.68 (12.55) | |
|  | Mean (SD) | 14.94 (7.29-22.78) | 14.29 (6.47-22.22) | 14.29 (6.28-22.70) | 13.85 (6-22.10) | 13.44 (5.62-21.58) | 12.74 (4.86-21.31) | 12.10 (4.87-21.09) | 11.76 (4.29 - 20.25) | 13.25 (5.34-21.74) | 12.43 (5-21.05) | |
| % patients seen for 12+ outpatient visits | Median (IQR) | 15.27 (20.75) | 14.92 (20.63) | 14.44 (20.32) | 14.08 (20.26) | 13.57 (20.07) | 12.55 (19.72) | 11.97 (19.50) | 11.62 (19.19) | 15.60 (22.29) | 13.99 (21.55) | |
|  | Mean (SD) | 5.40 (0.70-22.22) | 5.19 (0.71-20.83) | 4.90 (0.68-20.47) | 4.49 (0.49-19.07) | 4.26 (0.47-17.67) | 3.11 (0.29-15.09) | 3 (0.27-13.48) | 2.58 (0.17 - 13.11) | 5.35 (0.76-20.93) | 4.04 (0.60-17.45) | |
| # billing days | Mean (SD) | 165.01 (71.52) | 166.50 (72.10) | 166.39 (73.01) | 166.32 (72.80) | 165.83 (73.82) | 162.68 (73.46) | 164.88 (72.86) | 162.74 (74.85) | 177.71 (78.31) | 170.17 (76.50) | |
|  | Median (IQR) | 181 (117-218) | 183 (120-221) | 182 (119-222) | 185 (117-221) | 183 (112-223) | 178 (111-220) | 179 (115-221) | 177 (109-221) | 195 (120-236) | 186 (117-227) | |
| Saw <100 patients/year | (n %) | 647 (30.93) | 633 (29.70) | 638 (29.50) | 630 (28.81) | 627 (28.41) | 614 (27.56) | 599 (26.78) | 608 (26.64) | 681 (29.73) | 656 (28.05) | |

| Table A9. Service volume for specific diagnoses and psychiatrist patient population in British Columbia from 2012/13 to 2020/21. | | | | | | | | | | | |  |  |
| --- | --- | --- | --- | --- | --- | --- | --- | --- | --- | --- | --- | --- | --- |
| FY |  | 2012/13 | 2013/14 | 2014/15 | 2015/16 | 2016/17 | 2017/18 | 2018/19 | 2019/20 | 2020/21 | 2021/22 | |  |
| Total # psychotherapy visits | Mean (SD) | 927.3 (734.9) | 948.38 (775.26) | 958.44 (771.51) | 950.07 (772.85) | 957.78 (764.33) | 922.77 (728.58) | 887.89 (702.56) | 858.88 (693.97) | 896.42 (735.15) | 839.87 (697.51) | |  |
|  | Median (IQR) | 775.0 (377.0-1320.0) | 788.50 (358-1298) | 814 (369-1330) | 791.50 (366.50-1342) | 782.50 (352-1380) | 746 (361-1314) | 750 (359-1286) | 715 (329-1213) | 750 (336-1266) | 708.50 (304-1187) | |  |
| # virtual visits | Mean (SD) | 3.2  (30.5) | 5.75 (52.45) | 8.43 (70.57) | 9.99 (65.79) | 12.20 (65.45) | 15.68 (86.10) | 21.35 (113.52) | 43.51 (136.23) | 577.59 (657.23) | 459.45 (588.36) | |  |
|  | Median (IQR) | 0 (0-0) | 0 (0-0) | 0 (0-0) | 0 (0-0) | 0 (0-1) | 0 (0-2) | 0 (0-3) | 15 (1-42) | 409 (61-873) | 274.50 (27-675) | |  |
|  | **Service volume for specific diagnoses** | | | | | | | | | | | | |
| # mental health Dx visits | Mean (SD) | 1087.5 (917.3) | 1104.81 (940.62) | 1104.05 (928.70) | 1099.12 (924.84) | 1120.13 (905.38) | 1088.37 (858.81) | 1089.41 (883.24) | 1067.17 (878.48) | 1161.32 (1002.15) | 1117.80 (1002.71) | |  |
|  | Median (IQR) | 890.5  (416-1514) | 886.50 (393-1504) | 908.50 (409-1517) | 886.50 (413.50-1520.50) | 903 (457-1545) | 878 (434-1517) | 876 (431-1576) | 835 (413-1486) | 909 (426-1636) | 888 (407-1588) | |  |
| # substance use Dx visits | Mean (SD) | 84.2 (862.1) | 85.24 (894.62) | 80.95 (837.80) | 81.60 (763.39) | 95.75 (825.63) | 102.38 (804.39) | 111.72 (779.36) | 114.65 (807.74) | 111.57 (783.32) | 117.15 (828.58) | |  |
|  | Median (IQR) | 0 (0-11) | 0 (0-12) | 0 (0-11) | 0 (0-10) | 0 (0-11) | 0 (0-12) | 1 (0-14) | 0 (0-15) | 0 (0-14) | 0 (0-13) | |  |
| # Non-MH Non-SU Dx visits | Mean (SD) | 162.6 (432.6) | 163.34 (444.30) | 165.61 (425.10) | 173.76 (441.61) | 174.45 (425.14) | 165.97 (408.96) | 173.32 (424.14) | 178.20 (431.49) | 202.95 (518.74) | 203.15 (565.99) | |  |
|  | Median (IQR) | 27.5  (3-114) | 23 (3-109) | 24 (2-112) | 26 (2-134) | 24 (3-138) | 28 (3-127) | 26 (4-124) | 28 (3-137) | 27 (3-155) | 25 (3-145) | |  |
|  | **Patient population** | | | | | | | | | | | | |
| # visits for patients treated for SUD | Mean (SD) | 214.0 (940.8) | 225.86 (965.72) | 223.53 (890.01) | 236 (836.38) | 263.39 (905.12) | 270.85 (870.21) | 292.11 (846.49) | 293.29 (873.26) | 299.16 (853.47) | 306.87 (904.45) | |  |
|  | Median (IQR) | 71.5  (17-210) | 69.50 (16-223) | 73 (15-227) | 73 (20-249) | 79 (22-273) | 76 (20-288) | 94 (21-305) | 90 (21-297) | 91 (25-318) | 93.50 (20-341) | |  |
| # visits for patients treated for schizophrenia etc | Mean (SD) | 411.4 (542.4) | 423.94 (575.94) | 437.73 (583.57) | 445.91 (591.92) | 462.35 (611.91) | 468.51 (602.96) | 476.15 (604.41) | 473.77 (605.51) | 503.10 (685.28) | 508.08 (716.65) | |  |
|  | Median (IQR) | 182  (34-587) | 177.50 (33-593) | 186.50 (39-651) | 190.50 (38-655.50) | 201 (41-667) | 202 (39-705) | 223 (38-704) | 213 (34-693) | 224 (37-731) | 225.50 (37-760) | |  |

| Table A10. Service volume for specific diagnoses and psychiatrist patient population in Manitoba from 2012/13 to 2020/21. | | | | | | | | | | | |  |  |
| --- | --- | --- | --- | --- | --- | --- | --- | --- | --- | --- | --- | --- | --- |
| FY | |  | 2012/13 | 2013/14 | 2014/15 | 2015/16 | 2016/17 | 2017/18 | 2018/19 | 2019/20 | 2020/21 | | 2021/22 |
| Total # psychotherapy visits | | Mean (SD) | 426.65 (470.73) | 434.70 (478.72) | 430.26 (459.18) | 398.33 (440.78) | 378.22 (429.86) | 349.07 (426.51) | 340.75 (380.42) | 354.36 (390.18) | 358.72 (384.32) | | 311.33 (340.18) |
|  |  | Median (IQR) | 259 (84-564) | 275 (95-563) | 303 (97-547.50) | 261 (79.50-523.50) | 246 (61.50-527) | 211 (48-476) | 217.50 (60-490) | 246 (99-478) | 276 (112-453) | | 206 (80-420) |
| # virtual visits | | Mean (SD) | 0.33 (2.17) | 0.20 (1.65) | 0.23 (1.56) | 0.15 (1.06) | 0.27 (1.49) | 0.24 (1.55) | 0.53 (2.78) | 24.17 (28.62) | 609.38 (636.64) | | 514.33 (566.25) |
|  |  | Median (IQR) | 0 (0-0) | 0 (0-0) | 0 (0-0) | 0 (0-0) | 0 (0-0) | 0 (0-0) | 0 (0-0) | 16 (2-33) | 388 (176-815) | | 341.50 (147-652) |
|  | **Service volume for specific diagnoses** | | | | | | | | | | | | |
| # mental health Dx visits | | Mean (SD) | 1148.32 (1017.91) | 1116.55 (1017.75) | 1105.84 (1037.56) | 1085.16 (1001.95) | 1046.35 (957.76) | 1043.62 (984.21) | 1239.96 (1154.55) | 1284.43 (1181.68) | 1408.17 (1238.71) | | 1400.03 (1305.73) |
|  |  | Median (IQR) | 875 (355-1805) | 790 (318-1818) | 810.50 (294.50-1717) | 833 (316.50-1681.50) | 762 (313-1565.50) | 773 (269-1591) | 888 (356-1809) | 940 (392-1940) | 1051 (403-2325) | | 957 (365-2288) |
| # substance use Dx visits | | Mean (SD) | 14.35 (32.78) | 11.70 (28.29) | 11.58 (26.98) | 8.87 (18.59) | 7.22 (15.32) | 6.47 (16.87) | 12.67 (56.77) | 13.29 (105.34) | 13.69 (101.64) | | 9.90 (35.95) |
|  |  | Median (IQR) | 3 (0-17) | 3 (0-11) | 2 (0-11) | 1 (0-8.50) | 1 (0-7) | 1 (0-6) | 2 (0-10) | 1 (0-6) | 0 (0-5) | | 0 (0-6) |
| # Non-MH Non-SU Dx visits | | Mean (SD) | 137.83 (203.44) | 129.22 (208.47) | 129.23 (193.58) | 130.21 (204.11) | 119.45 (191.52) | 117.01 (193.49) | 157.77 (212.57) | 197.47 (366.53) | 206.08 (548.42) | | 194.53 (503.11) |
|  |  | Median (IQR) | 59  (10-163) | 34  (8-150) | 44.50  (6-173) | 42  (5.50-145) | 37  (5.50-142.50) | 38  (5-132) | 66.50  (11-232) | 75  (12-236) | 68  (12-188) | | 60  (12-181) |
|  | **Patient population** | | | | | | | | | | | | |
| # visits for patients treated for SUD | | Mean (SD) | 65.32 (94.92) | 61.26 (94.79) | 65.56 (100.26) | 63.36 (103.05) | 63.51 (108.40) | 70.24 (108.55) | 119.09 (211.96) | 133.69 (225.18) | 148.90 (248.79) | | 138.40 (203.44) |
|  |  | Median (IQR) | 31 (6-89) | 29  (3-75) | 25  (3.50-81.50) | 29  (2-75.50) | 29  (4.50-76) | 29  (4-85) | 40  (8-124) | 43  (11-158) | 52  (6-195) | | 47.50  (8-167) |
| # visits for patients treated for schizophrenia etc | | Mean (SD) | 354.79 (546.25) | 337.50 (534.19) | 321.53 (521.25) | 325.56 (537.93) | 326.72 (517.05) | 325.13 (525.47) | 482.22 (746.82) | 502.37 (759.35) | 522.97 (761.84) | | 545.29 (793.70) |
|  |  | Median (IQR) | 85 (10-471) | 82 (5-404) | 71.50 (10.50-374) | 78.50 (11.50-332.50) | 85 (16.50-331) | 77 (12-360) | 105 (13-600) | 113 (20-674) | 133 (17-726) | | 151.50 (21-727) |

| Table A11. Service volume for specific diagnoses and psychiatrist patient population in Ontario from 2012/13 to 2020/21. | | | | | | | | | | | | |  |
| --- | --- | --- | --- | --- | --- | --- | --- | --- | --- | --- | --- | --- | --- |
| FY | |  | 2012/13 | 2013/14 | 2014/15 | 2015/16 | 2016/17 | 2017/18 | 2018/19 | 2019/20 | 2020/21 | 2021/22 | |
| Total # psychotherapy visits | | Mean (SD) | 706.13 (692.59) | 710.85 (698.99) | 700.16 (700.96) | 693.37 (701.86) | 681.10 (701.59) | 658.07 (699.14) | 654.55 (692.75) | 624.25 (677.47) | 381.77 (568.43) | 374.69 (564.56) | |
|  |  | Median (IQR) | 516 (164.50-1057.50) | 522 (164-1073) | 498 (163-1058) | 480 (150-1053) | 471 (135-1019) | 438 (123-1012.50) | 429 (127-996) | 395 (113-935) | 117 (5-541) | 109 (4-532) | |
| # virtual visits | | Mean (SD) | 2.66 (19.60) | 4.45 (33.68) | 5.85 (43.19) | 9.07 (73.27) | 13.50 (117.97) | 17.99 (164.37) | 27.20 (213.37) | 61.37 (255.30) | 839.44 (971.84) | 744.30 (912.30) | |
|  |  | Median (IQR) | 0 (0-0) | 0 (0-0) | 0 (0-0) | 0 (0-0) | 0 (0-0) | 0 (0-0) | 0 (0-0) | 20 (2-51) | 590  (188-1208) | 510  (131-1036) | |
|  | **Service volume for specific diagnoses** | | | | | | | | | | | | |
| # mental health Dx visits | | Mean (SD) | 1231.86 (1151.44) | 1240.44 (1142.20) | 1235.80 (1148.55) | 1234.09 (1149.35) | 1224.11 (1144.20) | 1208.82 (1163.70) | 1206.39 (1150.34) | 1175.76 (1137.78) | 1237.06 (1166.35) | 1183.70 (1138.47) | |
|  |  | Median (IQR) | 966 (420-1668.50) | 971  (447-1692) | 957  (431-1705) | 972  (429-1674) | 962  (407-1688) | 943.50  (396-1628) | 938  (407-1607) | 902  (376-1579) | 961  (394-1725) | 926  (375-1629) | |
| # substance use Dx visits | | Mean (SD) | 56.01 (506.53) | 61.81 (601.46) | 64.83 (587.43) | 71.32 (578.81) | 71.46 (583.72) | 70.64 (572.28) | 72.64 (539.97) | 69.10 (526.73) | 71.63 (597.28) | 72.18 (561.44) | |
|  |  | Median (IQR) | 0 (0-11.50) | 0 (0-13) | 0 (0-13) | 0 (0-15) | 1 (0-15) | 1 (0-16) | 1 (0-20) | 1 (0-19) | 1 (0-18) | 1 (0-20) | |
| # Non-MH Non-SU Dx visits | | Mean (SD) | 86.34 (233.81) | 94.72 (261.12) | 96.10 (276.82) | 100.10 (290.41) | 98.33 (247.44) | 100.65 (247.05) | 108.86 (259.46) | 111.03 (262.12) | 117.97 (247.58) | 177.86 (351.61) | |
|  |  | Median (IQR) | 8 (0-69) | 10 (0-74) | 8 (0-73) | 10 (0-74) | 11 (0-77) | 11 (0-85.50) | 16 (0-93) | 16 (0-101) | 18 (0-116) | 33 (1-195) | |
|  | **Patient population** | | | | | | | | | | | | |
| # visits for patients treated for SUD | | Mean (SD) | 166.84 (547.52) | 177.06 (641.38) | 186.80 (633.88) | 200.53 (629.46) | 203.89 (639.54) | 210.50 (643.64) | 223.36 (628.77) | 223.26 (621.24) | 225.01 (674.29) | 226.07 (643.14) | |
|  |  | Median (IQR) | 48 (13-155) | 51 (13-166) | 50 (14-170) | 54 (15-185) | 59 (15-190) | 58 (16-195.50) | 62 (16-218) | 62 (15-220) | 63 (15-213) | 64 (15-224) | |
| # visits for patients treated for schizophrenia etc | | Mean (SD) | 332.90 (555.71) | 348.48 (573.68) | 351.09 (579.36) | 359.98 (587.91) | 360.45 (576.59) | 367.90 (599.33) | 379.61 (602.98) | 377.85 (605.77) | 393.80 (614.63) | 388.10 (596.79) | |
|  |  | Median (IQR) | 95 (19-390.50) | 102 (20-425) | 100 (20-429) | 103 (23-442) | 107 (23-438) | 109 (23-422.50) | 111 (24-442) | 118 (23-447) | 126 (25-456) | 120 (24-477) | |

Figure A1. Plots of median number of total visits averaged over two fiscal years, by psychiatrist sex/gender and years since graduation (MD) grouped into 10-year bands in British Columbia, Manitoba, and Ontario (Figure 2A-E)

|  | Females | Males |
| --- | --- | --- |
| BC | 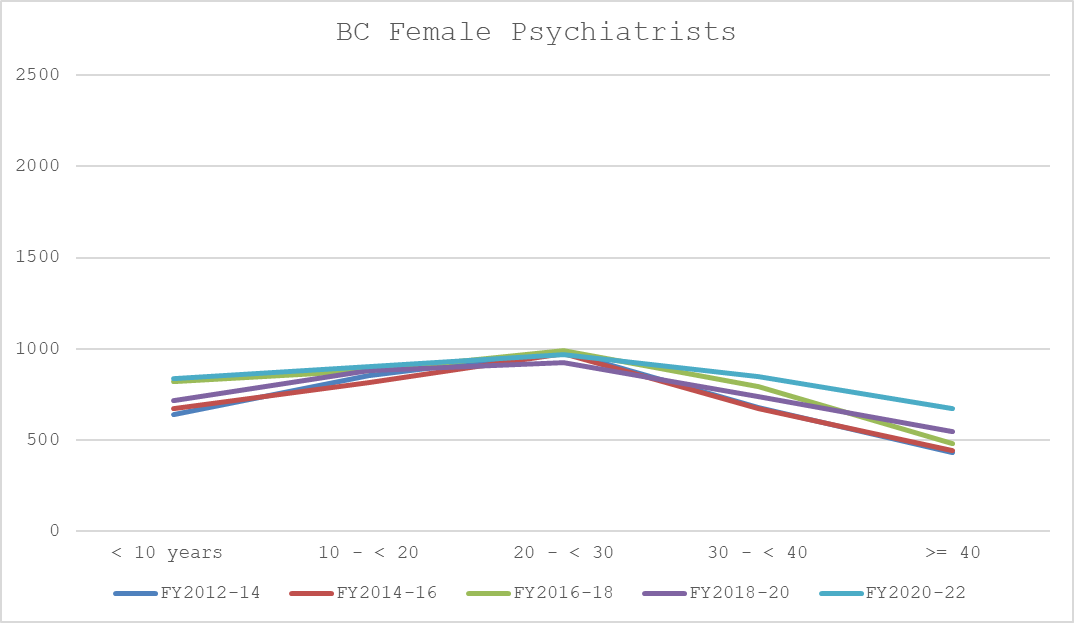 | 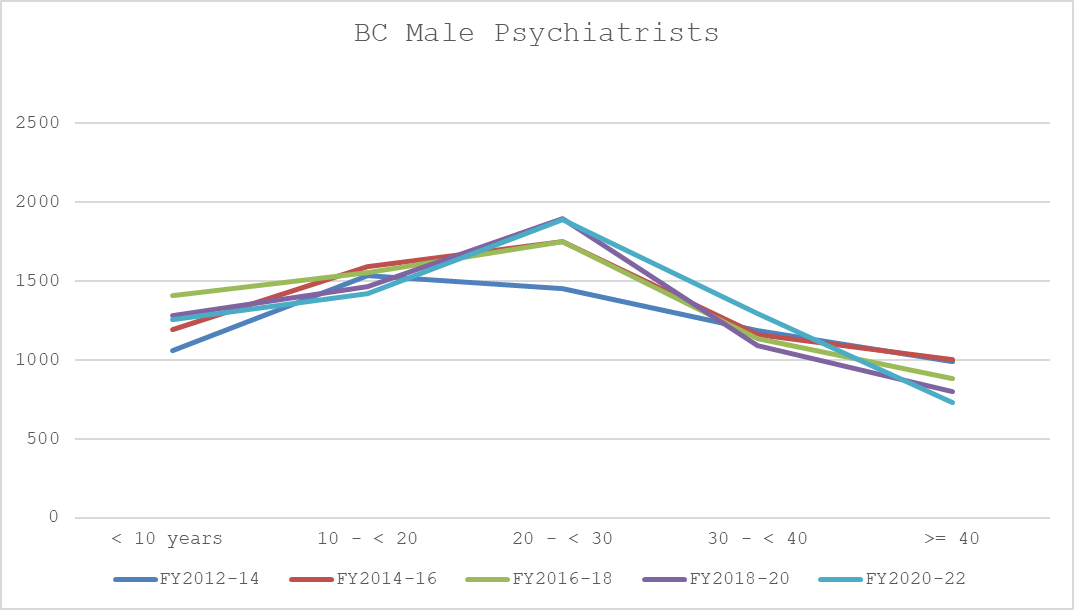 |
| MB | 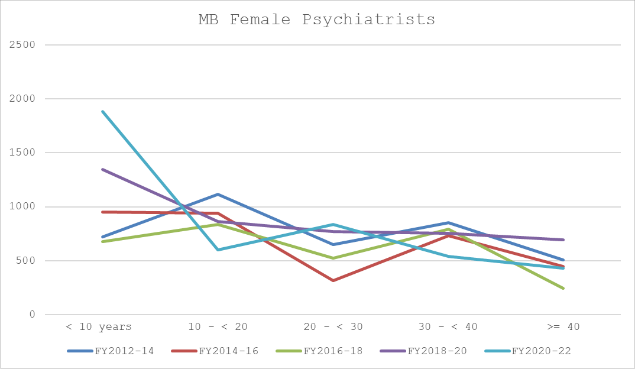 | 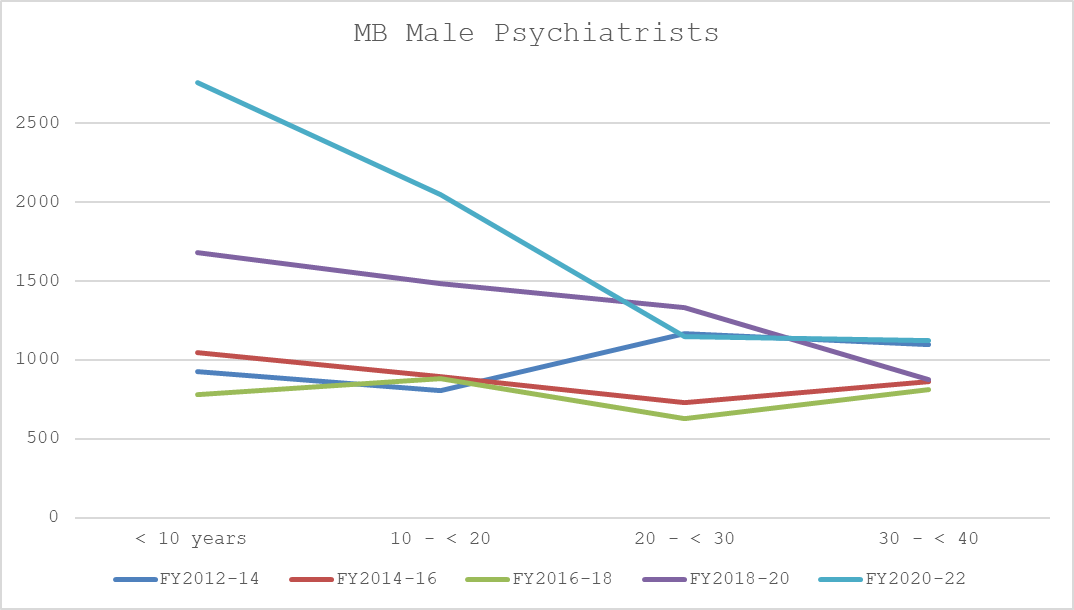 |
| ON | 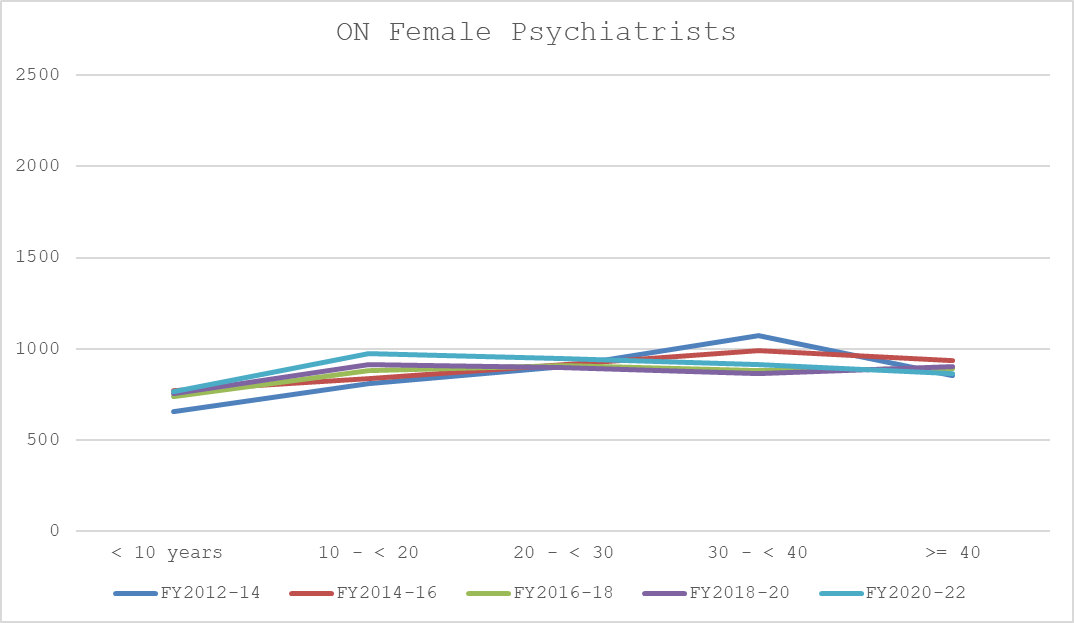 | 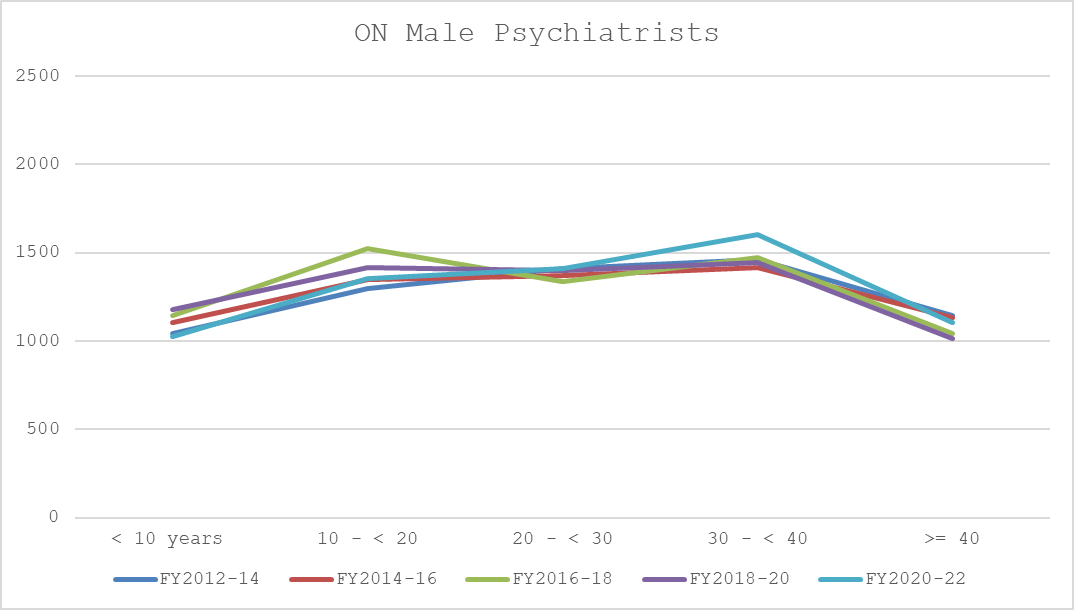 |

Figure A2. Median number of total visits according to psychiatrist sex/gender and number of years since graduation in each province from 2012/13 to 2021/22

|  | Females | Males |
| --- | --- | --- |
| BC | 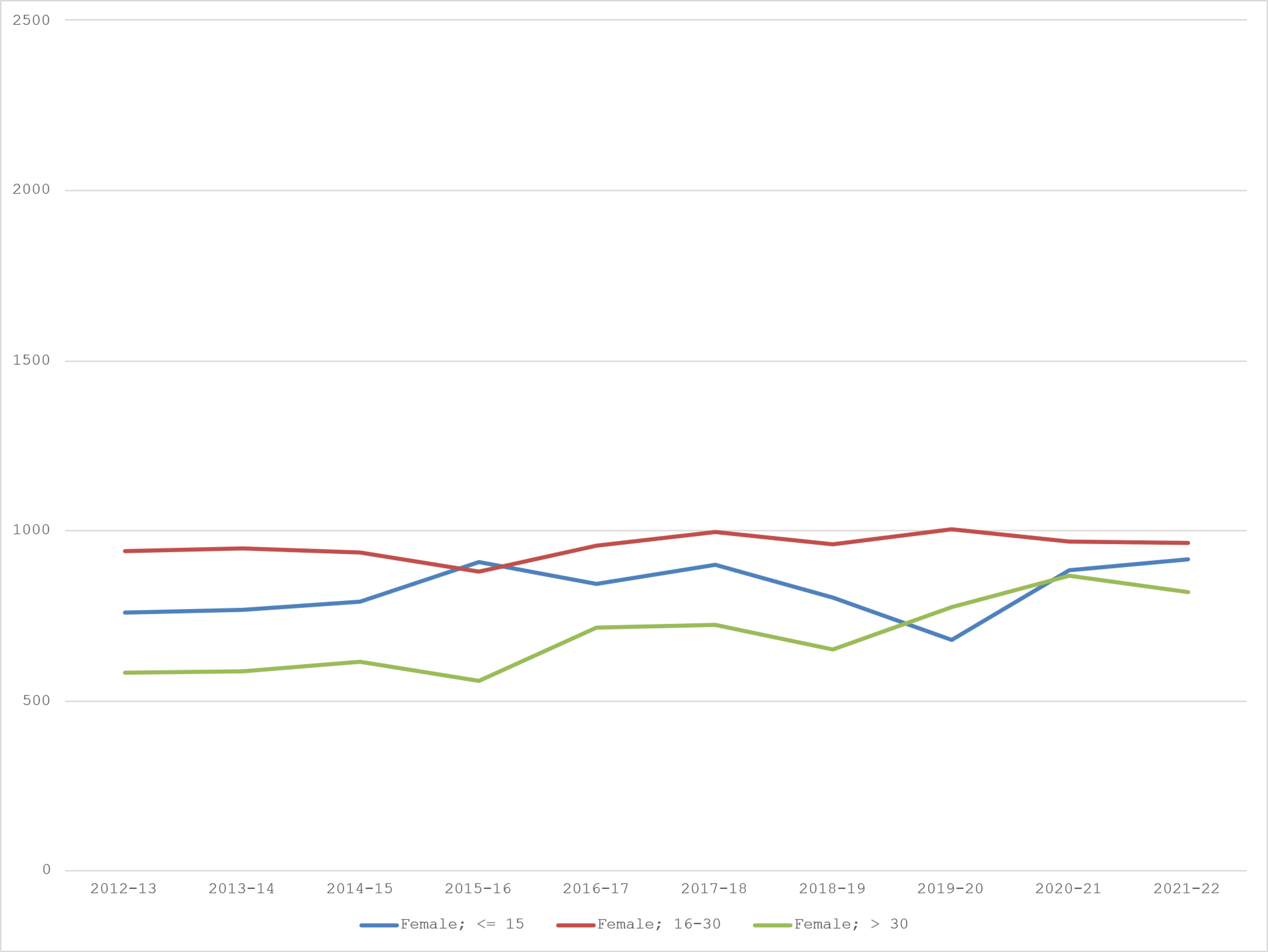 | 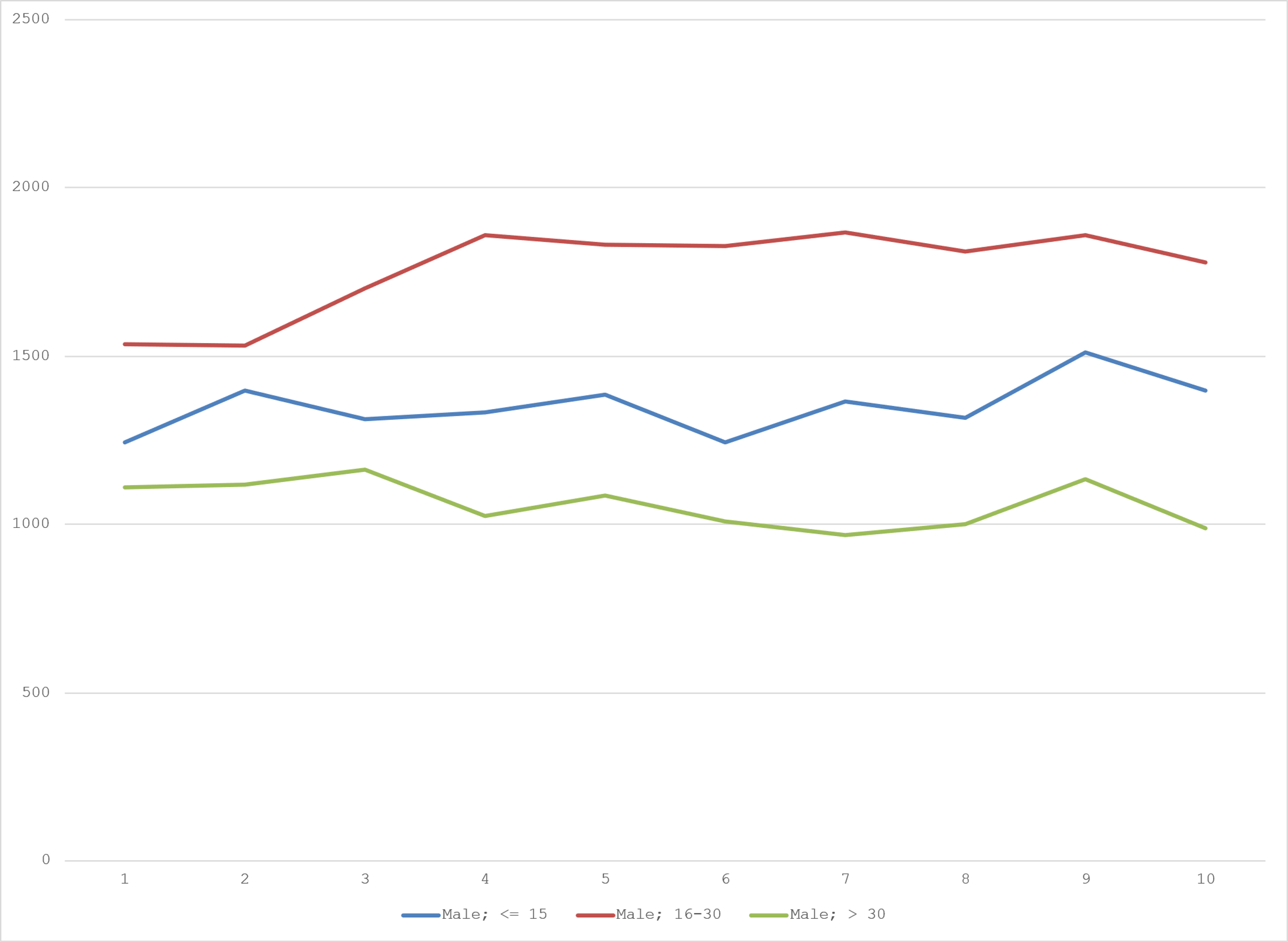 |
| MB | 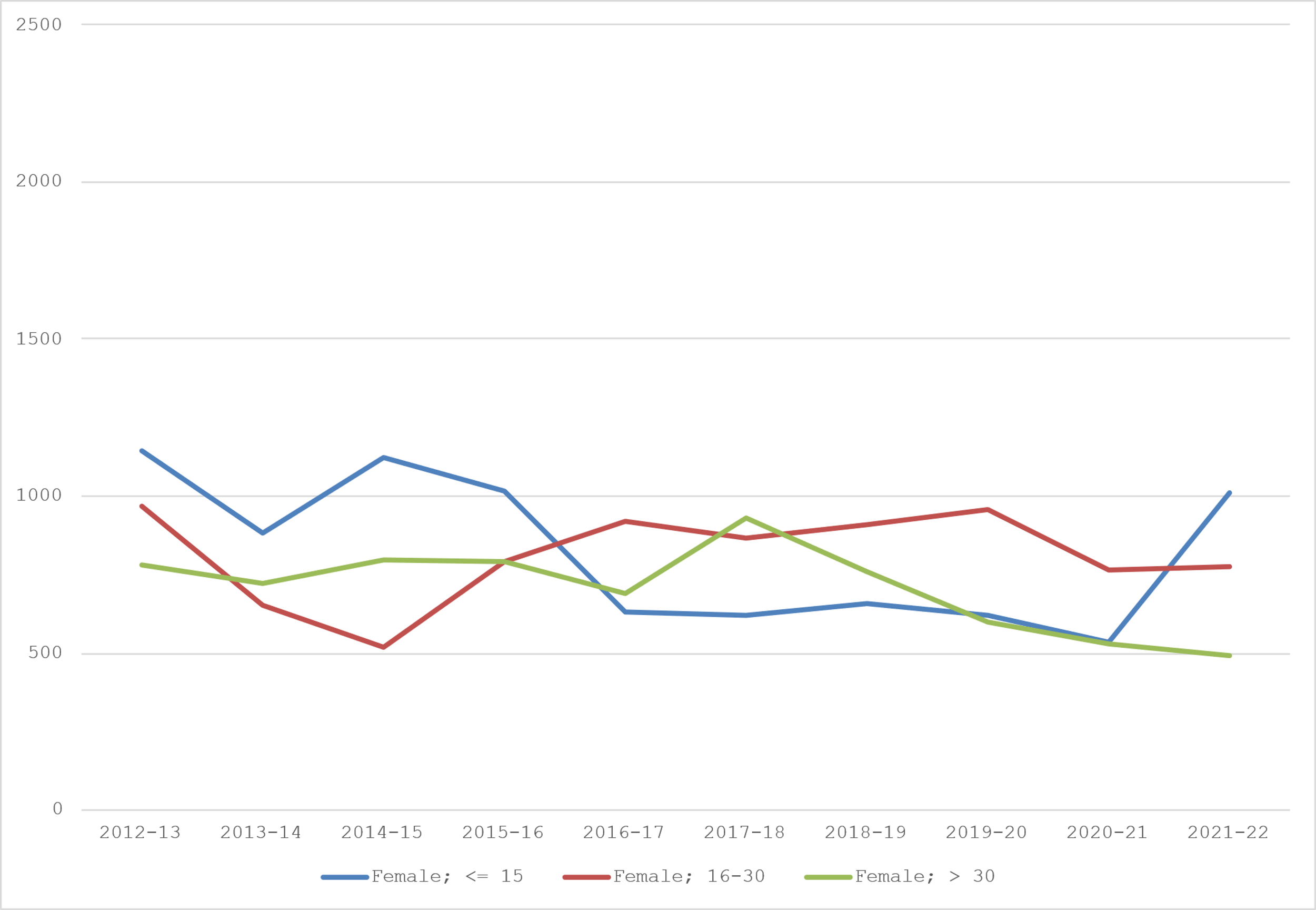 | 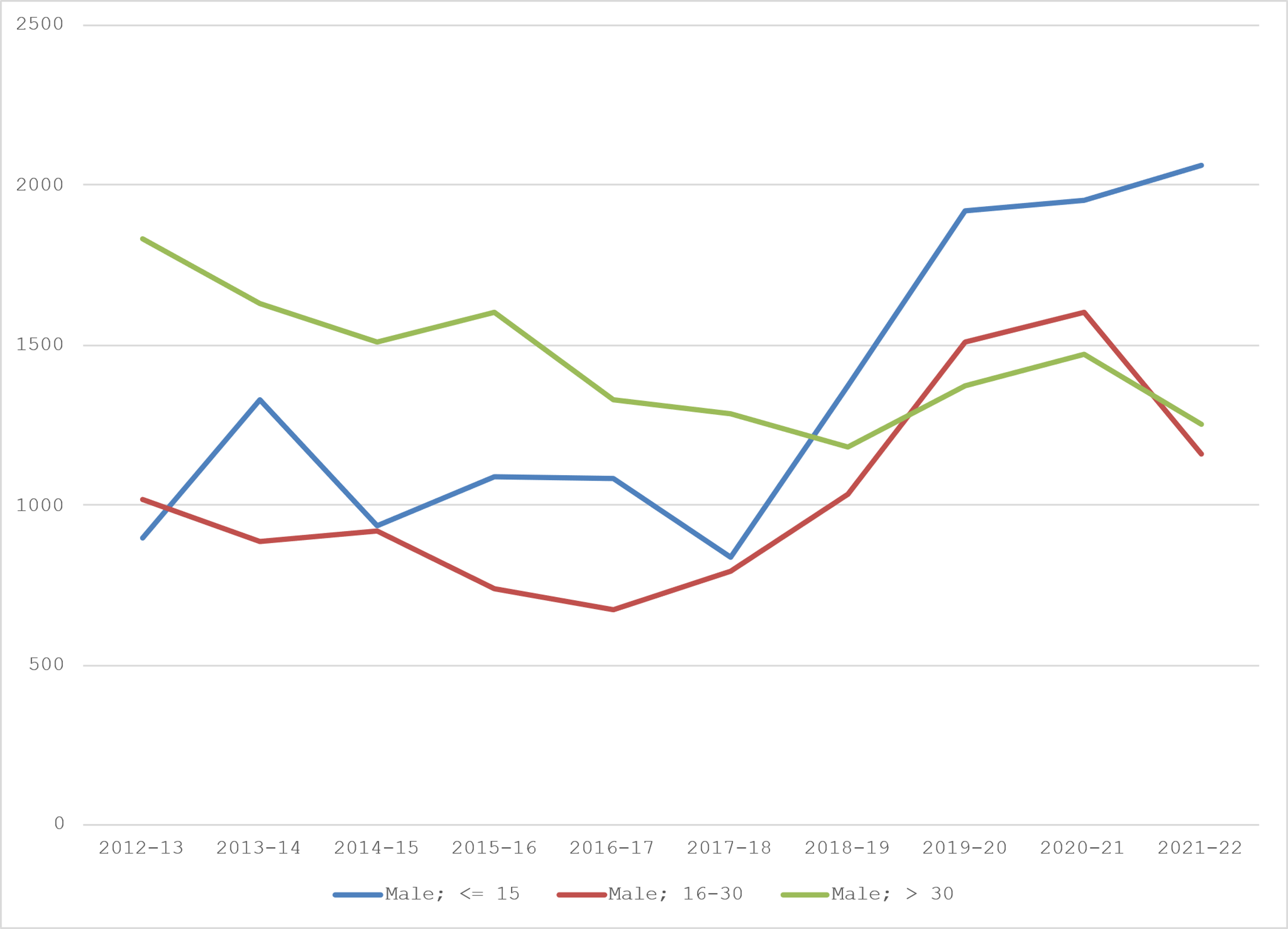 |
| ON | 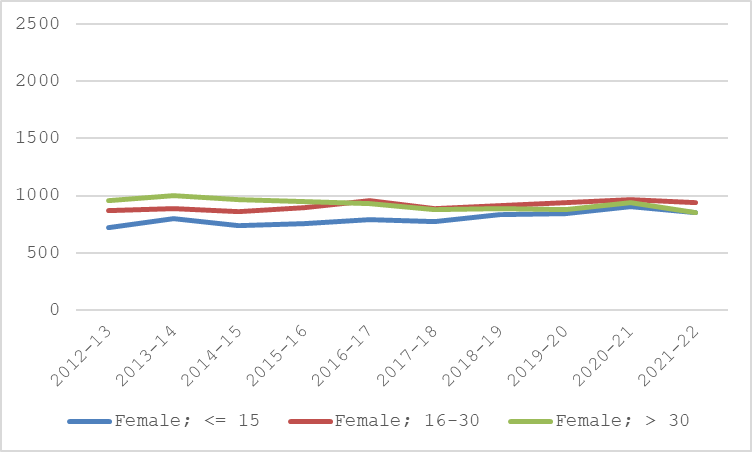 | 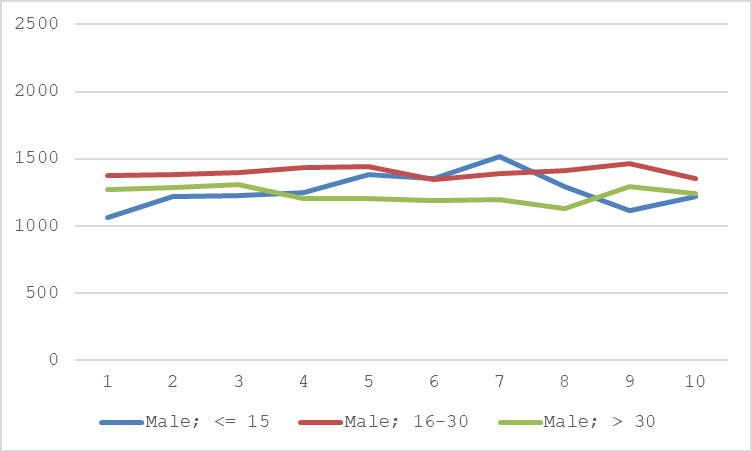 |
